# Antibody blockade of Jagged1 attenuates choroidal neovascularization

**DOI:** 10.1038/s41467-023-38563-w

**Published:** 2023-05-30

**Authors:** Torleif Tollefsrud Gjølberg, Jonas Aakre Wik, Hanna Johannessen, Stig Krüger, Nicola Bassi, Panagiotis F. Christopoulos, Malin Bern, Stian Foss, Goran Petrovski, Morten C. Moe, Guttorm Haraldsen, Johanna Hol Fosse, Bjørn Steen Skålhegg, Jan Terje Andersen, Eirik Sundlisæter

**Affiliations:** 1 Department of Immunology, Oslo University Hospital Rikshospitalet, 0372 Oslo, Norway; 2 Institute of Clinical Medicine and Department of Pharmacology, University of Oslo and Oslo University Hospital, 0372 Oslo, Norway; 3 Center of Eye Research, Department of Ophthalmology, Oslo University Hospital and University of Oslo, 0450 Oslo, Norway; 4 Department of Pathology, Oslo University Hospital Rikshospitalet, 0372 Oslo, Norway; 5 Department of Nutrition, Division of Molecular Nutrition, Institute of Basic Medical Sciences, University of Oslo, 0372 Oslo, Norway; 6 Department of Pediatric Surgery, Oslo University Hospital Rikshospitalet, 0372 Oslo, Norway

**Keywords:** Growth factor signalling, Antibody therapy, Macular degeneration

## Abstract

Antibody-based blocking of vascular endothelial growth factor (VEGF) reduces choroidal neovascularization (CNV) and retinal edema, rescuing vision in patients with neovascular age-related macular degeneration (nAMD). However, poor response and resistance to anti-VEGF treatment occurs. We report that targeting the Notch ligand Jagged1 by a monoclonal antibody reduces neovascular lesion size, number of activated phagocytes and inflammatory markers and vascular leakage in an experimental CNV mouse model. Additionally, we demonstrate that Jagged1 is expressed in mouse and human eyes, and that Jagged1 expression is independent of VEGF signaling in human endothelial cells. When anti-Jagged1 was combined with anti-VEGF in mice, the decrease in lesion size exceeded that of either antibody alone. The therapeutic effect was solely dependent on blocking, as engineering antibodies to abolish effector functions did not impair the therapeutic effect. Targeting of Jagged1 alone or in combination with anti-VEGF may thus be an attractive strategy to attenuate CNV-bearing diseases.

## Abstract (web summary)

Current treatment for neovascular age-related macular degeneration (nAMD) does not help all patients. Here, the authors show that using antibodies to block Jagged1 reduces disease burden in a model of nAMD, which could enable new treatment options.

## Introduction

Age-related macular degeneration (AMD) is a leading cause of vision loss worldwide. There are two types of AMD; the “dry” and the “wet” forms. Wet, or neovascular AMD (nAMD), is usually preceded by dry AMD, and is characterized by choroidal neovascularization (CNV) breaching the choroid-retinal barrier. This causes leakage and hemorrhaging into the retina, leading to photoreceptor death. CNV is responsible for the majority of severe vision loss in AMD and may progress to legal blindness if left untreated^1^. 8.7% of the global population aged between 45-85 years have AMD to date, of which ~5% have the wet form^2^. While the underlying mechanisms of nAMD development are unclear, inflammation is a critical component, and CNV lesions are infiltrated by immune cells such as T cells and phagocytes^3–6^.

Treatment of nAMD with anti-vascular endothelial growth factor (VEGF) antibody-based therapeutics by intravitreal (IVT) injections reduces neovascularization, inflammation, edema, and bleeding^7^. However, some patients show no or only limited therapeutic effect to anti-VEGF treatment^8,9^. Also, the effect can decline with time, and continuous VEGF blockade may even be toxic to ocular cell types and detrimental to retinal tissue, as VEGF maintains homeostatic functions in ocular physiology^10–14^. As nAMD is a chronic disease requiring sometimes lifelong monitoring and treatment, this is a problematic aspect of anti-VEGF-based treatment. Thus, there is a need for alternative treatment options based on identification of new targets or the use of combinatorial treatment to enhance the efficacy of VEGF blockade^8,15,16^.

Vascular endothelial cells are central in angiogenesis and the initiation and amplification of inflammatory responses. Even though their role in recruiting leukocytes to inflammatory lesions is well characterized, control of their response to inflammatory mediators and angiogenic factors is incompletely understood^17^. Importantly, the Notch signaling pathway enables inflammation through interaction with the NF-κB pathway^18^, and results in production of pro-inflammatory cytokines, like tumor necrosis factor alpha (TNF-α), interleukin-1 beta (IL-1β) and Toll-like receptor agonists. As such, inhibition of Notch signaling ameliorates experimental arthritis, acute colitis, acute lung injury and graft-versus-host disease^19^. At the cellular level, Notch signaling affects the functions of both endothelial and immune cells and, can act pro- or anti-angiogenic in a context-dependent manner^20–22^. For example, Notch ligand Delta-like 4 (DLL4) inhibits tip cell formation during VEGF-induced sprouting angiogenesis and promotes endothelial cell quiescence^23–25^. Like DLL4, Jagged1 is a membrane-bound protein with epidermal growth factor (EGF)-like repeats^26^. However, Jagged1 acts as an unambiguous pro-angiogenic factor through its relation to DLL4^25,27^, and as such stimulates angiogenesis during development^25^, wound-healing^28^ and tumor angiogenesis^29^. Thus, Notch ligands are promising therapeutic targets in several cancer types^30^.

Here, we report that a monoclonal antibody targeting Jagged1 reduces CNV development in an experimental mouse model by decreasing lesion size, the numbers and reactivity of associated mononuclear phagocytes and the levels of pro-inflammatory cytokines. Furthermore, we demonstrate that Jagged1 expression in human endothelial cells is independent of VEGF stimuli. When we simultaneously targeted Jagged1 and VEGF by monoclonal antibodies in the CNV mouse model, the effect surpassed that of sole targeting of either factor. At last, we show that the therapeutic effect of targeting Jagged1 is solely dependent on target blockade, as engineering for lack of antibody effector functions did not impair the effect. Hence, we reveal that Jagged1 is an attractive target in CNV pathogenesis, which can be targeted alone or in combination with anti-VEGF to attenuate CNV-bearing retinal disorders.

## Results

### Rationale for targeting Jagged1

**Fig. 1:**
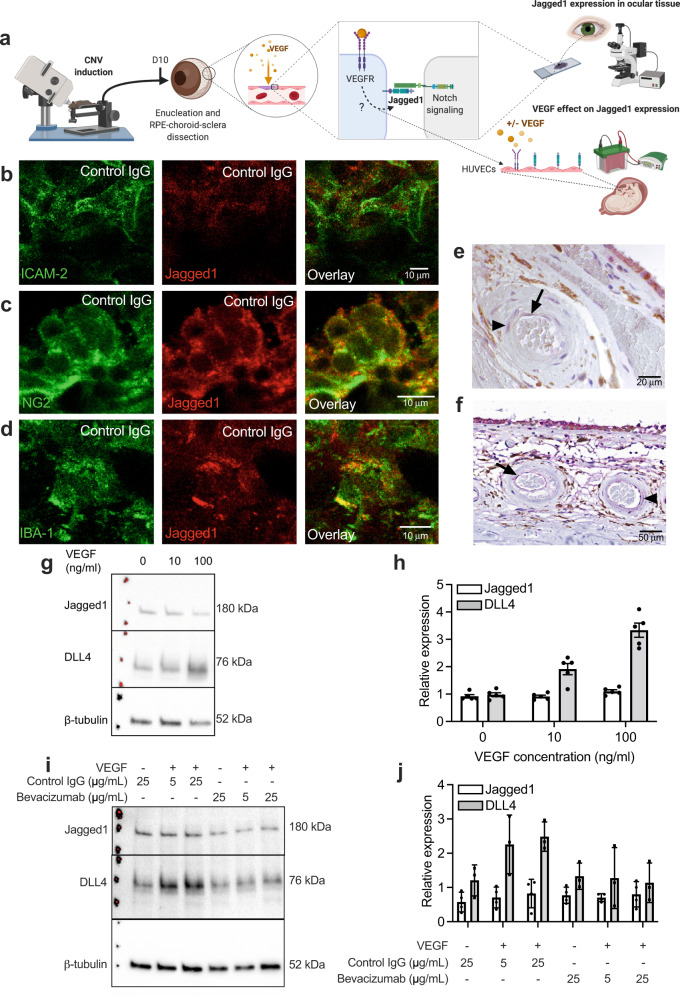
Jagged1 is expressed in ocular blood vessels, pericytes and monocytes, and is unaffected by VEGF stimuli in HUVECs. **a** Illustration of methodology. CNV was induced at day 0 by laser treatment of C57BL/6 J mice. At day 10, eyes were enucleated and dissected into RPE/choroidal-sclera flat mounts. Separately, HUVECs were stimulated with VEGF in culture. The illustration was created with Biorender.com. **b**–**d** Immunostaining of RPE/choroidal-sclera flat mounts following CNV induction in C57BL/6 J mice, showing colocalization of Jagged1 (red) with green ICAM-2 (**b**), NG2 (**c**), and IBA-1 (**d**). Images in **b**–**d** were taken with a confocal microscope using the appropriate excitation and emission filters for each fluorophore. **e** Immunostaining of ocular tissue with dry age-related maculopathy and pigment changes. Immunoreactivity is observed in endothelial cells of choroidal arteries (arrow) and in smooth muscle cells lining the vessel (arrowhead). **f** Immunostaining of ocular tissue from a normal human eye. As in **e**, both endothelial cells of choroidal arteries (arrow) and surrounding smooth muscle cells (arrowhead) display immunoreactivity. Immunostaining procedures yielding representative images shown in **b**–**f** were performed twice in individual experiments. **g**–**j** HUVECs cultured for 48 hours and starved for 24 hours after seeding, before treatment with **g**, **h** increasing concentrations of human VEGF and/or **i**, **j** bevacizumab or control IgG for 24 hours. Images in **g**, **i** show representative western blots showing Jagged1, DLL4, and β-tubulin in HUVECs under the specified conditions. Graphs in **h**, **j** show the quantification of bands exemplified in **g**, **i**, displaying relative intensity of Jagged1 and DLL4 when normalized against β-tubulin. Data are means ± SEM representing measurements from three individual experiments. Source data are provided as a Source Data file.

We hypothesized that Jagged1 is a relevant target in nAMD. This was based on several lines of evidence. First, we identified Jagged1 in mouse eyes in a relevant disease setting. This was done by taking advantage of a mouse model of nAMD where CNV was induced by laser injury of C57BL/6 J mice (Fig. 1a). Jagged1 staining was clear in CNV lesions on RPE-choroid-sclera flat mounts from mice 10 days after laser treatment, where it co-localized with the endothelial cell marker intercellular adhesion molecule-2 (ICAM-2; Fig. 1b), neuron-glial antigen 2 (NG2) on pericytes (Fig. 1c)^31^ and ionized calcium-binding molecule-1 (IBA-1) on macrophages (Fig. 1d)^15,31–33^. All antibodies used for staining are listed in Supplementary table 1.

Second, to address the translatability of Jagged1 in an ocular setting, we showed by immunohistochemistry on postmortem human eyes with anatomical signs of dry AMD by means of subretinal drusen deposits and retinal pigment epithelium (RPE) changes, that Jagged1 is occasionally expressed by the inner endothelial cell layer of choroidal blood vessels. In addition, endothelial staining of Jagged1 was detected in vessels of different sizes in choroidal vascular layers, but also in the middle and outer layers of arteries (Fig. 1e). Similar immunoreactivity was observed in normal ocular tissue of human eyes (Fig. 1f).

Third, we assessed the relationship between Jagged1 expression and VEGF signaling by stimulating confluent, serum-starved human umbilical vein endothelial cells (HUVECs) with VEGF (Fig. 1g-j). While DLL4 is induced by VEGF as a negative feedback regulator in endothelial cells^23,34^, Jagged1 is rather upregulated by inflammatory cytokines^21^. Protein expression was addressed by Western blotting, which revealed that indeed, VEGF stimuli increased DLL4 expression (Fig. 1g-h). This response was VEGF-specific, evident by its absence in the presence of the VEGF-blocking monoclonal antibody bevacizumab (Fig. 1i, j). Notably, increasing concentrations of VEGF did not affect Jagged1 expression under any of the tested conditions (Fig. 1g-j). This was also the case when performing without serum-starvation (Supplementary Fig. 1), demonstrating that the findings were not affected by growth factors in the culturing medium. Thus, the data support that Jagged1 expression in endothelial cells is independent of VEGF signaling.

Taken together, this provided a rationale for therapeutic targeting of Jagged1 in the laser-induced CNV disease model.

### Anti-JAG1 inhibits CNV formation and reduces vascular leakage

**Fig. 2:**
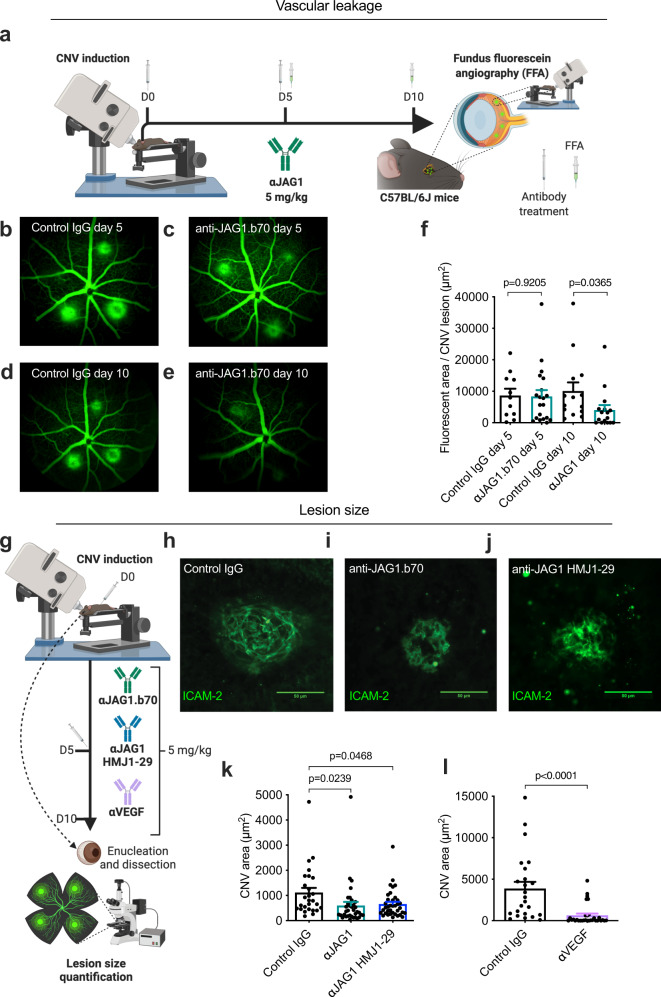
Antibody targeting of Jagged1 reduces laser-induced choroidal neovascularization and vascular leakage. **a** Illustration of methodology. Antibodies were administered as indicated; after laser injury and at day 5. Vascular leakage was measured by fluorescein angiography performed at days 5 and 10 after CNV induction while laser scars visualized by immunofluorescence 10 days after photocoagulation in RPE-choroid-sclera flat mounts. Endothelial cells were labeled with rat anti-mouse ICAM-2. The illustration was created with Biorender.com. **b**–**e** Representative late phase (13 min) fluorescein angiograms of **b**, **d** control IgG-treated or **c**, **e** anti-JAG1 treated mice (5 mg/kg) 5 days (**b**, **c**) or 10 days (**d**, **e**) after CNV induction. **f** Mean leakage areas measured 5 or 10 days after CNV induction. Anti-JAG1 reduced vascular leakage at day 10 (*n* = 6 eyes from six individual mice per bar (two-tailed, unpaired Student’s *t* test). **g** Illustration of methodology. Antibodies were administered as indicated, after laser injury, and at day 5, and day 10, eyes were enucleated and lesion size quantified by microscopy analysis on RPE/choroidal-sclera flat mounts. The illustration was created with Biorender.com. **h**–**j** Representative images of CNV in **h** control-, **i** anti-JAG1.b70 treated mice or **j** anti-JAG1 HMJ1-29-treated mice. **k** Quantification of lesion size following treatment with 5 mg/kg of either control antibody, anti-JAG1 or anti-JAG1 HMJ1-29 *(n* = 10 eyes from 6 individual mice for the control IgG group, 11 eyes from 6 individual mice for the anti-JAG1.b70 group, and 12 eyes from 6 individual mice for the anti-JAG1 HMJ1-29 group. (one-way ANOVA, Dunnett’s multiple comparison test). Jagged1 targeting significantly reduced CNV area. **l** Significant reduction in lesion size following treatment with 5 mg/kg anti-VEGF (*n* = 10 eyes from 6 individual mice for the control IgG group, and 12 eyes from 6 individual mice in the anti-VEGF group). Bar graphs in **f**, **k**, and **l** show mean values and SEM (two-tailed, unpaired Student’s *t* test). Source data are provided as a Source Data file.

To study the effects of Jagged1 targeting in the laser-induced CNV mouse model, we used a phage-display selected mouse immunoglobulin G2a (mIgG2a) antibody (anti-JAG1.b70^35^, hereafter referred to as anti-JAG1). A monoclonal mIgG2a with irrelevant specificity was used as a control. 5 mg/kg of each of antibody was injected intraperitoneally (IP) after laser treatment at day 0 (D0) and day 5 (D5) (Fig. 2a). The central parameters to assess treatment efficacy in the CNV model are reduction in vascular leakage and lesion size. Vascular leakage was assessed by fundus fluorescein angiography (FFA), performed using a Micron IV imaging system at D5 and D10 (Fig. 2a). Treatment with anti-JAG1 significantly reduced the fluorescent area 10 days after laser injury, while the control antibody did not (Fig. 2b-f). As scar size may correlate with fluorescent area, this also indicated an effect of anti-JAG1 on lesion size.

Next, we sought to determine whether targeting Jagged1 can reduce CNV lesion size (Fig. 2g–k). Mice were euthanized at D10, and following enucleation and dissection, CNV area was measured by immunofluorescence staining for ICAM-2, which is the most effective means of identifying CNV in this model^36^. ICAM-2 was clearly detected in lesions (Fig. 2h–j). Anti-JAG1 suppressed laser injury-induced CNV, resulting in a ~40% reduction in CNV area compared to the antibody control (Fig. 2k). This phenotype was also seen using another anti-mouse Jagged1 monoclonal antibody from hamster (Fig. 2k; clone HMJ1-29^37^). As expected, repeating the experiment with an anti-mouse VEGF mIgG2a antibody (anti-VEGF B20-4.1.1, referred to as anti-VEGF hereafter) reduced CNV size by 80% (Fig. 2l). In conclusion, Jagged1 blockade actively reduced lesion size after laser-induced CNV formation.

### Jagged1 blockade reduces production of inflammatory mediators

Jagged1 expressed by endothelial cells has been shown to be a potent pro-angiogenic regulator^25^. In the context of the laser-induced CNV model, inflammatory cells, and in particular mononuclear phagocytes, are known to be potent initiators of the angiogenic process partly through their capacity to release pro-angiogenic factors^38–40^. Therefore, we assessed how anti-JAG1 treatment affected the inflammatory profile following CNV induction.

**Fig. 3:**
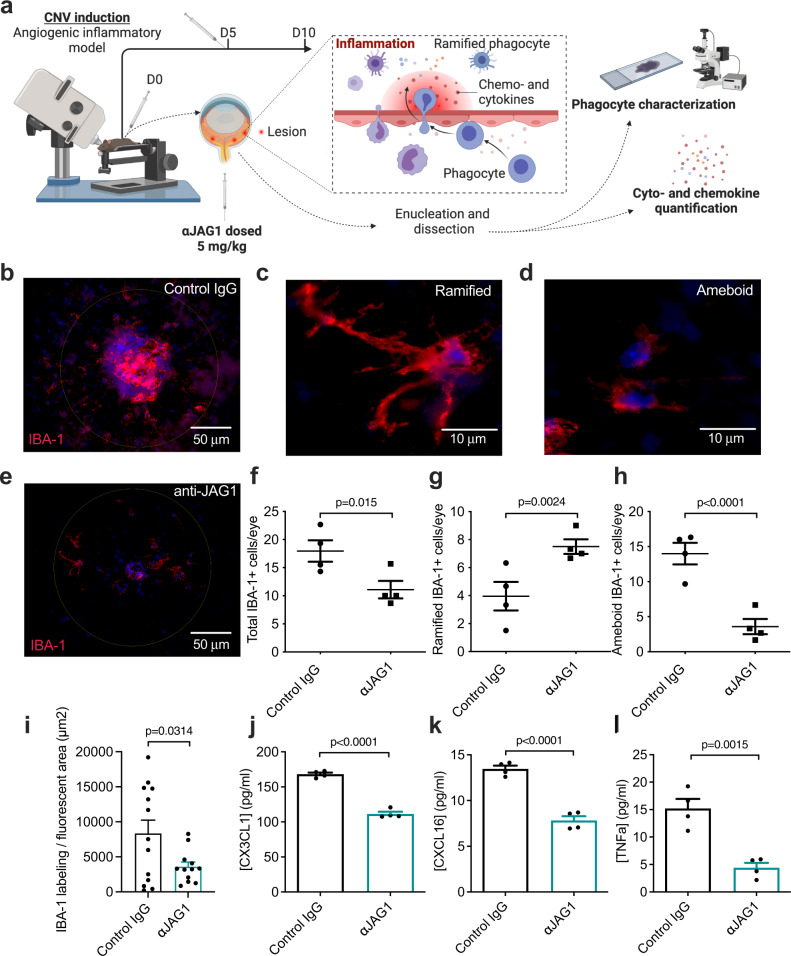
Antibody targeting of Jagged1 ameliorates microgliosis and reduces inflammatory traits in an angiogenic inflammation model. **a** Illustration of methodology. Antibodies were administered as indicated after laser injury and at day 5. At day 10, eyes were enucleated, dissected, and immunostained for phagocyte characterization in RPE/choroidal-scleral flat mounts. Eyes used for cyto- and chemokine quantification assays were enucleated at day 4. The illustration was created with Biorender.com. **b**, **e** Representative IBA-1 staining of RPE/choroidal flat mounts detecting microglia/macrophages in laser spots 10 days after laser coagulation in control mice (**b**) or anti-JAG1 treated animals (**e**). IBA-1 is shown in red. Circular selection diameter: 200 µm. **c**, **d** Representative images of ramified (**c**) and ameboid (**d**) microglia/macrophages in lesions such as shown in **b** and **e**. **f**–**h** Quantification of total (**f**), ramified- (**g**) or ameboid-shaped (**h**) mononuclear phagocytes in laser spots. Values show mean ± SEM (*n* = 4 eyes from 4 individual mice per column; calculated from a total of 12 and 13 CNV lesions for control IgG and anti-JAG1, respectively (two-tailed, unpaired Student’s t-test). **i** IBA-1 positive area in images used for cell counting quantified in ImageJ (two-tailed, unpaired Student’s *t* test). **j**–**l** Concentrations of cyto- and chemokines in the retina of mice treated with either control IgG or anti-JAG1 were determined with a multiplex assay 4 days after CNV induction. Retinal concentrations of the pro-inflammatory CX3CL1 (**j**), CXCL16 (**k**), and TNFa (**l**) in the presence of anti-JAG1. Data are means ± SEM of duplicate determinations for two pooled samples (three retinas were pooled as one sample) and are the same as those shown in Supplementary Fig. 3a, b (two-tailed, unpaired Student’s *t* test). Source data are provided as a Source Data file.

Fluorescence microscopy analysis on RPE-choroid-sclera flat mounts was used to assess the number of IBA-1-positive mononuclear phagocytes within and around the CNV lesion (Fig. 3a). This revealed clear IBA-1 staining in lesions after treatment with control IgG (Fig. 3b). We addressed the phagocytic profile by counting the total number of IBA-1 positive cells in lesions, and by characterizing their activation status by classifying them as either ramified or ameboid. While resting mononuclear cells show a distinct ramified morphology (Fig. 3c), activated cells retract their protrusions and acquire an ameboid shape (Fig. 3d^39^). After Jagged1 blockade, IBA-1 staining was reduced (Fig. 3e), which was mirrored by a significant 60% reduction in total number of IBA-1 positive cells (Fig. 3f). Furthermore, Jagged1 blockade resulted in a 2.0-fold higher fraction of ramified cells (Fig. 3g), and a 3.9-fold lower fraction of ameboid cells (Fig. 3h). To address the objectivity of these findings, the total area of IBA-1 specific fluorescence in lesions was quantified in image J, which confirmed a significant reduction after Jagged1 blockade (Fig. 3i). Manual quantifications were confirmed by independent counting and characterization of IBA-1 positive cells by a second observer (Supplementary Fig. 2a–c).

Next, we used a multiplex assay to assess the levels of pro-inflammatory cytokines and chemokines in the retina and choroid-sclera 4 days after laser treatment. Anti-JAG1 reduced retinal concentrations of the inflammatory chemokines CX3CL1 (fractalkine) and CXCL16 along with the pro-inflammatory cytokine TNF-α, indicating an anti-inflammatory effect of Jagged1 blockade (Fig. 3j–l). Concentrations of various pro-inflammatory cytokines and chemokines in the retina and choroid-sclera are shown in Supplementary Fig. 3.

**Fig. 4:**
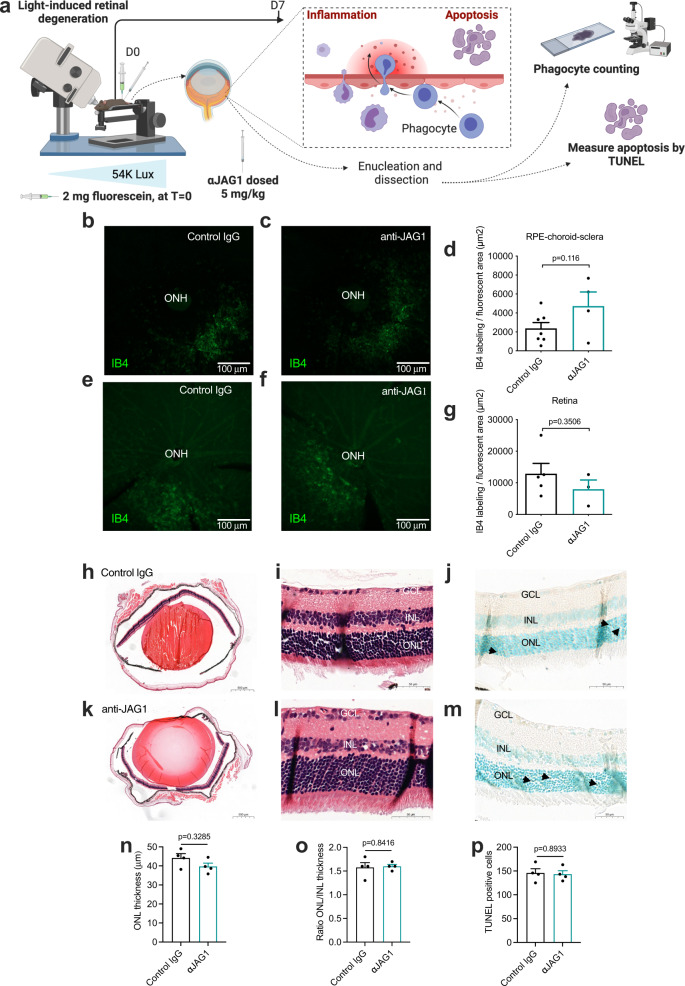
Antibody targeting of Jagged1 does not reduce light-induced retinal degeneration (LIRD). **a** Illustration of methodology. At day 0, mice were injected with fluorescein (at time (*T*) = 0) 3 minutes prior to a 4-minute exposure to blue light (54,000 Lux) in the right eye (starting at *T* = 3 minutes), followed by a repositioning and light exposure in the left eye (starting at *T* = 10 minutes). After light exposure, antibodies were administered as indicated. At day 7, eyes were enucleated and either dissected into retinal or choroid-sclera flat mounts for either monocyte characterization (right eyes), or used for apoptotic measurements by TUNEL assay (left eyes). The illustration was created with Biorender.com. **b**, **c** IB4-staining in RPE/choroidal-sclera flat mounts centered on the optic nerve head (ONH) in control mice (**b**) or anti-JAG1 treated animals (**c**). **d** Quantification of IB4-positive area in flat-mounts exemplified in **b**, **c**. Data are means ± SEM and represents 4 eyes from 4 individual mice treated with anti-JAG1 and 7 eyes from 7 individual mice treated with control IgG (two-tailed, unpaired Student’s *t* test). **e**, **f** IB4-staining in retinal flat-mounts in control mice (**e**) or anti-JAG1 treated animals (**f**). **g** Quantification of IB4-positive area in flat-mounts exemplified in **e** and **f**. Data are means ± SEM and represents 3 eyes from 3 individual mice treated with anti-JAG1 and 5 eyes from 5 individual mice treated with control IgG (two-tailed, unpaired Student’s *t* test). **h**–**j** Representative images from control animals after LIRD, showing **h**; hematoxylin and eosin (H&E) stained) whole eye, the neuroretina with nuclei stained in blue using hematoxylin (**i**) showing the ganglion cell layer (GCL), inner nuclear layer (INL), and the outer nuclear layer (ONL), and the neuroretina stained with TUNEL assay (**j**). Arrowheads indicate TUNEL positive/apoptotic cells. **k**–**m** Representative images from animals treated with anti-JAG1. **n**–**p** Quantification of (**n**) ONL thickness and (**o**) ratio of ONL/INL thickness in retinas exemplified in **i** and **l** and (**p**) TUNEL-positive cells exemplimfied in **j**, **m**. Data are means ± SEM of triplicate analysis of three eyes from three individual mice per column (two-tailed, unpaired Student’s *t* test). Source data are provided as a Source Data file.

To understand whether the reduction in CNV-associated mononuclear phagocytes was a direct anti-inflammatory effect or a consequence of reduced neovascularization, we tested anti-JAG1 in a non-angiogenic eye model of inflammation—the light-induced retinal degeneration model (LIRD; Fig. 4a)^41^. In this model, isolectin B4 was used as a phagocytic marker. This was deemed a feasible approach, as staining for isolectin B4 together with IBA-1 in both retinal and RPE-sclera flat mounts demonstrated intense colocalization in non-vessel structures (Supplementary Fig. 2d–f). Although isolectin B4 is largely employed to identify endothelial cells, it also detects activated microglia both in rodents and humans^42^. Because the isolectin B4 staining gave such a strong signal intensity and vessels could easily be discerned from other cells, we chose it for quantification of signal intensities in images taken at low (×10) magnification. There was a tendency towards more microglia in RPE-choroid-sclera complexes (Fig. 4b, d) and less in retinal flat mounts after anti-JAG1-treatment (Fig. 4e–g), but no significant difference between anti-JAG1-treated mice and control mice was observed (Fig. 4d, g).

A consequence of light-induced retinal damage is apoptosis and a decrease in the number of cells over the entire retina, in particular the outer nuclear layer (ONL)^43^. We therefore measured and compared ONL thickness, including the ONL/inner nuclear layer (INL) ratio to normalize ONL thickness given the variations that may occur due to obliquity in tissue sectioning, as well as TUNEL quantification. Briefly, this revealed similar results for control IgG-treated (Fig. 4h–j) and anti-JAG1-treated mice (Fig. 4k–m), with no significant differences (Fig. 4n–p).

To corroborate our findings, we also tested anti-JAG1 in the delayed-type hypersensitivity model for human allergic contact dermatitis (Supplementary Fig. 2g–k)^44^. This is a model for an acute inflammatory response in the ear skin, characterized by dermal edema and leukocyte infiltration. Anti-JAG1 inhibited ear swelling at 24 hours (Supplementary Fig. 2h, i), which could reflect an effect on vascular permeability, but we observed no significant difference in the number of IBA-1-positive cells between anti-JAG1-treated mice and control mice (Supplementary Fig. 2j, k). Together, these findings suggest that Jagged1 blockade neither protects against light-induced photoreceptor cellular apoptosis nor directly affects mononuclear phagocyte recruitment and/or activation.

### Jagged1 expression in CNV lesions and retinal vascular morphology is not affected by Jagged1 blockade

**Fig. 5:**
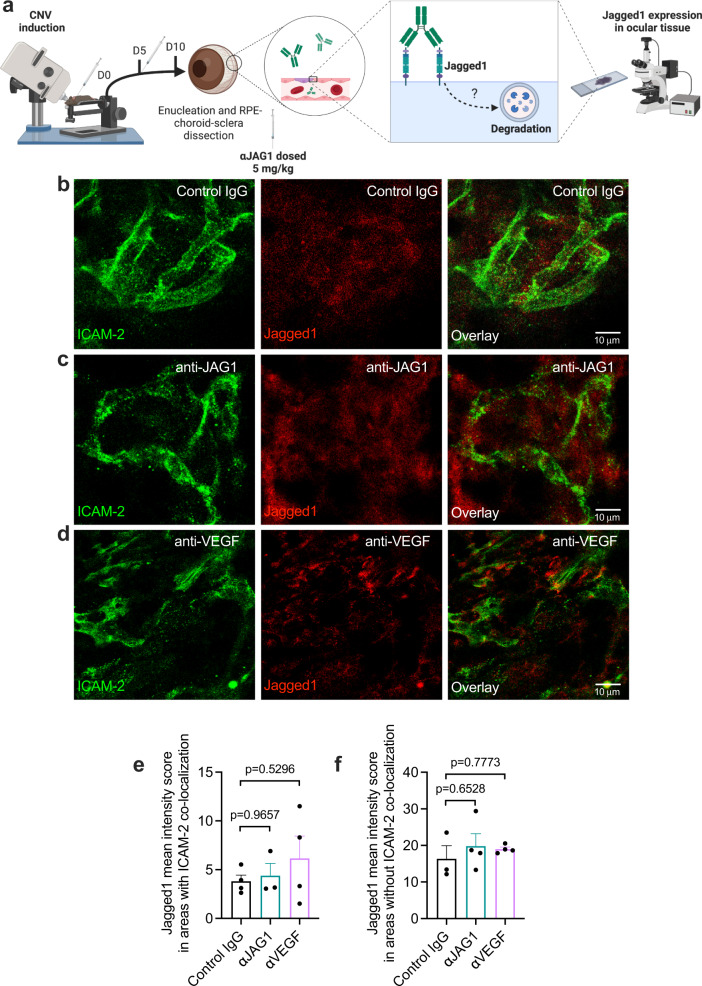
Antibody targeting of Jagged1 does not alter target expression. **a** Illustration of methodology. Antibodies were administered as indicated at days 0 and 5. Eyes were enucleated at day 10 and Jagged1 expression analyzed by confocal microscopy in RPE/choroidal-sclera flat mounts. The illustration was created with Biorender.com. **b**–**d** Immunostaining of RPE/choroidal-sclera flat mounts following CNV induction, showing ICAM-2 (green) and Jagged1 (red) in mice treated with **b** control IgG, **c** anti-JAG1 or **d** anti-VEGF. **e** Quantification of ICAM-2 – Jagged1 mean intensity score in sections exemplified in **b**–**d**. **f** Jagged1 mean intensity score in areas without ICAM-2 colocalization in images exemplified in **b**–**d**. Data represents means ± SEM of three eyes in total from three individual mice per treatment. Data in **e**, **f** were compared by one-way ANOVA with Šídák’s multiple comparisons test. Source data are provided as a Source Data file.

Therapeutic targeting of the Notch signaling pathway may have toxic effects on target-expressing tissues^45^. While this effect is notably less prominent for ligand-specific Notch modulation than for receptor or pan-Notch blockade^45,46^, antibody-induced target degradation or toxicity to retinal tissues could be a hurdle for clinical implementation of Jagged1 blockade in nAMD. To address this, we visualized the amount of Jagged1 found in CNV lesions after antibody treatment (Fig. 5a). Briefly, we found that neither the control IgG (Fig. 5b), anti-JAG1 (Fig. 5c) nor anti-VEGF (Fig. 5d) affected the colocalization of the endothelial marker ICAM-2 and Jagged1. Quantification revealed no significant alterations of Jagged1 levels after antibody treatment relative to control IgG (Fig. 5e, f).

Next, we addressed the effects of Jagged1 blockade on retinal vascular morphology and endothelial cell proliferation (Supplementary Fig. 4). By staining retinal flat-mounts with isolectin B4 (Supplementary Fig. 4a–c) and analyzing the resulting images with AngioTool Software (Supplementary Fig. 4d–f), we found that neither Jagged1 nor VEGF blockade significantly altered retinal vascular morphology. Furthermore, neither of the antibody treatments impaired endothelial cell proliferation, as evident by crystal violet staining of HUVEC cultures in both the presence and absence of VEGF stimuli (Supplementary Fig. 4m, n). Interestingly, addressing cytotoxicity by means of measuring release of lactate dehydrogenase (LDH) in culture revealed that in the presence of VEGF, anti-VEGF but not anti-JAG1 increased cytotoxicity of HUVECs (Supplementary Fig. 4o).

Furthermore, as we previously found that not only ocular endothelial cells, but also pericytes express Jagged1, we investigated the effects of Jagged1 blockade on pericyte coverage of retinal vessels (Supplementary Fig. 5). We did this by visualizing the extent and vicinity of isolectin B4 (marking endothelial cells) and NG2 (marking pericytes) in retinal flat-mounts from mice treated with either control IgG (Supplementary Fig. 5a) or anti-JAG1 (Supplementary Fig. 5b). Quantification revealed that Jagged1 blockade did not significantly affect pericyte coverage (Supplementary Fig. 5c).

Taken together, these findings strongly suggest that the observed effects of Jagged1 blockade in the CNV model are due to target blockade, not degradation, and that blockade is not toxic to retinal vascular tissue.

### Jagged1 blockade results in upregulation of DLL4 in endothelial cells in CNV lesions

The physiological effects of Jagged1 signaling are closely linked to the opposing Notch ligand DLL4^25^, forming a ligand pair with pathological implications in retinal disorders such as diabetic retinopathy^47^.

As a general rule, DLL4/Notch signaling restricts sprouting angiogenesis and promote endothelial quiescence^23–25^.

**Fig. 6:**
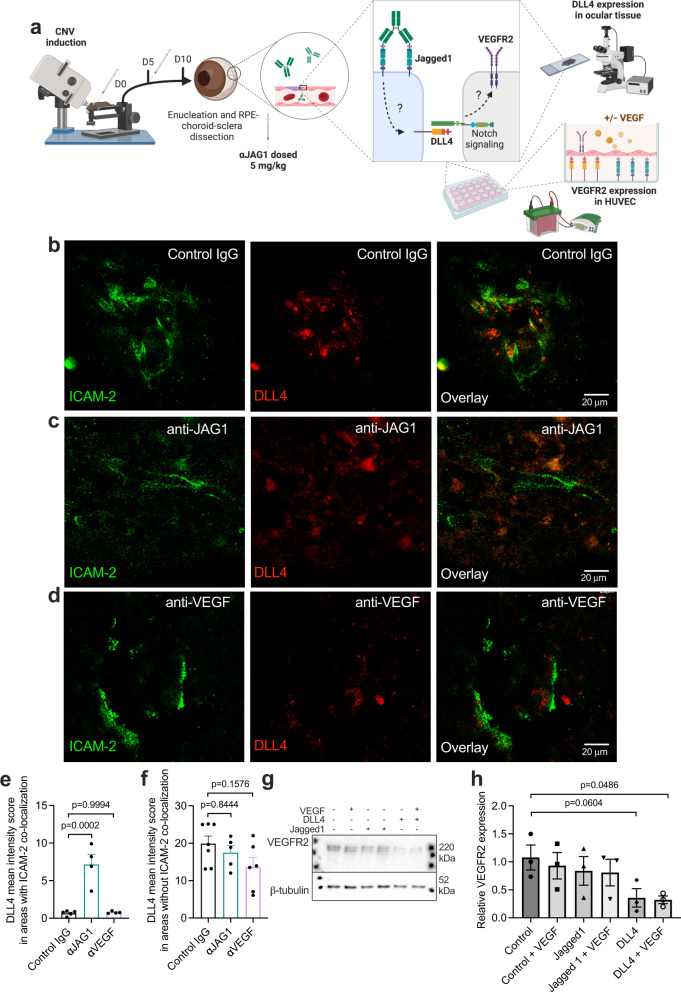
Antibody targeting of Jagged1 results in upregulation of DLL4 in vivo. **a** Illustration of methodology. Antibodies were administered as indicated at days 5 and 10, before enucleation and analysis of DLL4 expression in RPE/choroidal-sclera flat mounts by confocal microscopy. Separately, HUVECs were cultivated in wells coated with directionally captured DLL4 or Jagged1. Following stimulation with VEGF, the cellular expression of VEGFR2 was analyzed by Western blot. The illustration was created with Biorender.com. **b**–**d** Immunostaining of RPE/choroidal-sclera flat mounts following CNV induction, showing ICAM-2 (green) and Dll4 (red) in mice treated with **b** control IgG, **c** anti-JAG1 or **d** anti-VEGF. **e** Quantification of ICAM-2 – Dll4 mean intensity score in sections exemplified in **b**–**d**. Data represents means ± SEM of from 3 individual mice per treatment. **f** DLL4 mean intensity score in exclusively non-ICAM-2 co-localizing areas in images exemplified in **b**–**d**. **g** Western blot analysis of VEGFR2 expression and β-tubulin in HUVECs grown on either DLL4 or Jagged1, in the absence or presence of VEGF stimuli. **h** Quantification in ImageLab 4.1 of VEGFR2 bands exemplified in **f**, normalized against β-tubulin. Data represent means ± SEM of three individual experiments per column. Data in **e**, **f**, **h** were compared by one-way ANOVA with Šídák’s multiple comparisons test. Source data are provided as a Source Data file.

To address whether the observed effects of Jagged1 blockade could be related to DLL4 signaling, we visualized DLL4 expression in CNV lesions following antibody treatment (Fig. 6a). By immunohistochemical staining for ICAM-2 and DLL4, we found that DLL4 localizes to endothelial cells in control mice (Fig. 6b). Jagged1 blockade resulted in a markedly higher signal for DLL4 (Fig. 6c). A similar increase was not seen after VEGF blockade (Fig. 6d). Quantification of DLL4 and ICAM-2 positive area revealed a 7-fold upregulation of DLL4 after Jagged1 blockade, relative to control IgG (Fig. 6e). Interestingly, this increase in DLL4 was specific to endothelial cells, as similar effects were not observed in areas without ICAM-2 expression (Fig. 6f). Instead, anti-JAG1 and, to a larger extent, anti-VEGF caused a tendency towards reduced DLL4 expression in non-endothelial cells.

To understand whether these effects could relate to the VEGF response that drives nAMD, we next addressed the effects of Jagged1- and DLL4 signaling on the VEGF response in endothelial cells. We did this by capturing Jagged1 and DLL4 in an oriented manner in a cell culture plate before culturing HUVECs in the presence or absence of VEGF, then analyzing the cellular expression of VEGFR2 by Western blot (Fig. 6g). This demonstrated that in the presence of VEGF, DLL4 signaling reduced VEGFR2 levels by ~70% (Fig. 6h).

VEGF is a potent driver of vascular permeability, and as such, DLL4-mediated desensitization to VEGF could reduce edema following Jagged1 blockade. Alternatively, Jagged1 blockade could alleviate vascular leakage by increasing the expression of cellular junction proteins, such as VE-cadherin and ZO-1. To address this, we visualized VE-cadherin (Supplementary Fig. 6) and ZO-1 (Supplementary Fig. 7) in CNV lesions following antibody treatment. In general, VE-Cadherin and ZO-1 immunoreactivity was faint or barely detectable in all CNV lesions. We observed no apparent difference in neither VE-Cadherin nor ZO-1 expression levels or distributions between control IgG-, anti-JAG1- or anti-VEGF-treated mice.

Taken together, these findings suggest that an upregulation of DLL4 in endothelial cells, and consequently a desensitization of endothelial tissue to VEGF, could be part of the mechanism by which Jagged1 blockade elicit its effects in CNV.

### Dosing of anti-JAG1 for persistent circulatory antibody levels

**Fig. 7:**
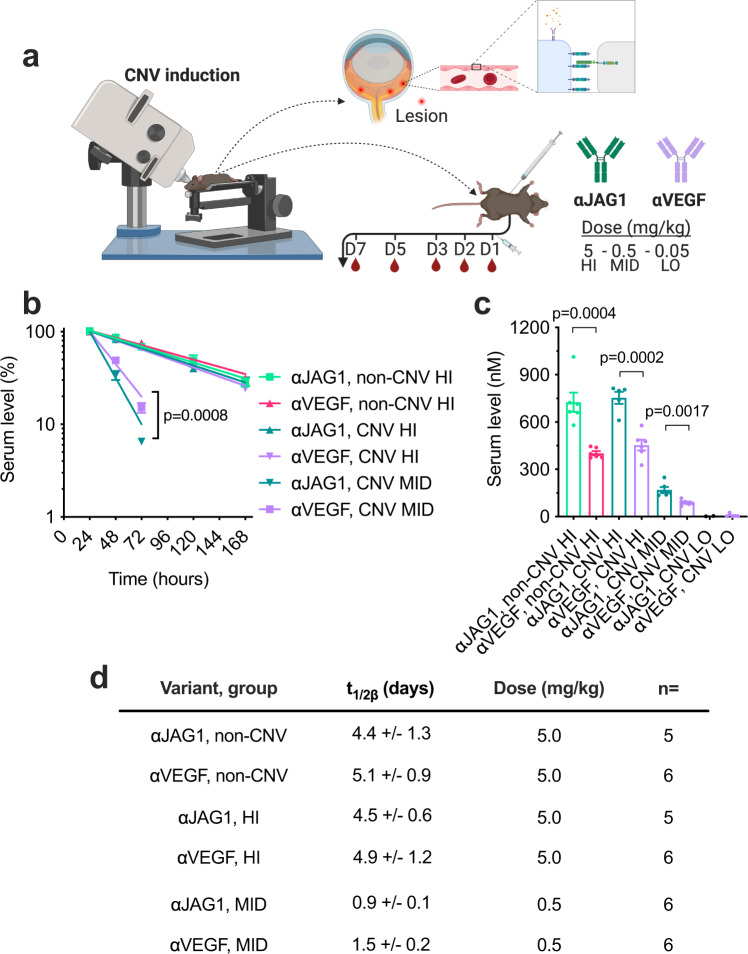
Circulatory properties of antibodies targeting Jagged1 and VEGF. **a** Illustration of methodology. Antibodies were administered as indicated, at day 0, either in non-laser-treated mice or after CNV induction. Antibody concentrations were determined in blood samples obtained at the indicated timepoints. The illustration was created with Biorender.com. **b** Log-linear decrease in plasma levels (signified by the solid lines, calculated by regression; *R*^2^ < 0.8 in all shown cases) of anti-JAG1 and anti-VEGF dosed at 5 mg/kg (HI) with or without (non-CNV) laser treatment and 0.5 mg/kg (MID) with laser treatment. **c** Bar graph showing averaged molar amounts and standard deviation of anti-JAG1 and following dosage of 5 mg/kg in mice with no laser treatment, and 5 and 0.5 mg/kg in laser-treated mice 24 hours after injection. **d** Table of calculated plasma half-life values, with the number of individual mice in each group (n) indicated. Graphs **b** and **c** show ± SEM, and values in the table (**d**) show mean values ± SD (two-tailed, unpaired Student’s *t* test). Source data are provided as a Source Data file.

In contrast to soluble VEGF, Jagged1 is membrane-bound and expressed in several tissues^26^. As such, it may act as an antigen-sink upon antibody injection, which would require tailoring of antibody dosing for long-term therapeutic effect^48^. To assess this, we measured the plasma half-life of anti-JAG1 and anti-VEGF through a dose titration range of 5.0 (HI), 0.5 (MID), and 0.05 (LO) mg/kg in laser-treated C57BL/6 J mice (Fig. 7a). A control group of untreated mice injected with the HI dosage was included. The results revealed that the plasma half-lives of anti-JAG1 and anti-VEGF were comparable, both in the presence and absence of induced CNV at 5.0 mg/kg (4.4 vs 5.1 days in non-CNV, respectively, 4.5 vs 4.9 days following laser treatment) (Fig. 7b, d). In contrast, at 0.5 mg/kg, the plasma half-life was estimated to be ~1.5-fold shorter for anti-JAG1 than anti-VEGF (Fig. 7b, d). Furthermore, anti-JAG1 was present at 1.7-1.9-fold higher amounts than anti-VEGF after 24 hours in the both the HI and MID groups, while in the LO groups we could only detect anti-VEGF at levels <5 nM (insufficient for half-life calculation) (Fig. 7c). Thus, a larger amount of anti-JAG1 was cleared from circulation following the initial distribution phase. Taken together, the data suggest that at lower dosages, anti-JAG1 has a higher clearance rate than anti-VEGF, in line with an antigen-sink effect, which can be overcome at higher dosages.

### Combined Jagged1 and VEGF targeting enhances therapeutic outcome

**Fig. 8:**
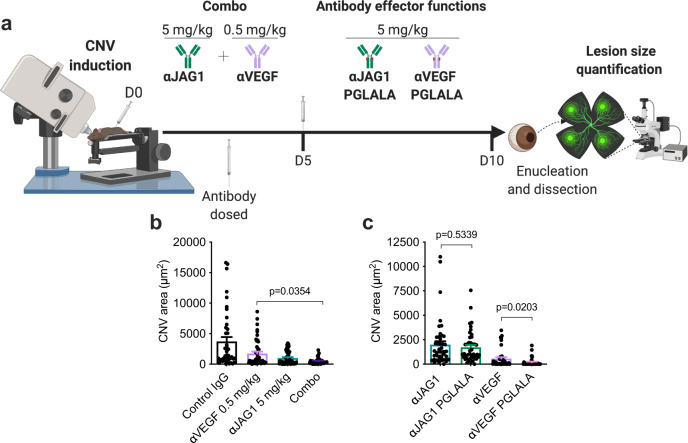
Therapeutic effect of antibody targeting of Jagged1 is additive to anti-VEGF and independent of antibody effector functions. **a** Illustration of methodology. Antibodies were administered following CNV induction as indicated, at days 0 and 5. Effector-negative antibodies were generated as PGLALA variants. At day 10, eyes were enucleated and lesion size in RPE/choroidal-sclera flat mounts was quantified. The illustration was created with Biorender.com. **b** Lesion size quantification following treatment with either 0.5 mg/kg anti-VEGF, 5 mg/kg anti-JAG1 or both antibodies combined compared to 5 mg/kg control antibody (*n* = 10 individual mice per group (one-way ANOVA with Šídák’s multiple comparisons test). **c** Quantification of lesion size following treatment with 5 mg/kg of WT and effector-negative PGLALA-engineered anti-VEGF and anti-JAG1 mIgG2a antibodies (*n* = 8 individual mice per group (two-tailed, unpaired Student’s *t* test). Values from lesion size quantification (**b**, **c**) show mean ± SEM. Source data are provided as a Source Data file.

Based on the finding that VEGF signaling is independent of Jagged1 expression, we investigated whether simultaneous blockade could elicit additive effect in the CNV model. Due to the dramatic effect of 5 mg/kg anti-VEGF (Fig. 2l), we reduced the dosage to 0.5 mg/kg and gave it in combination with 5 mg/kg anti-JAG1 at both D0 and D5 (Fig. 8a). This was chosen as a feasible approach, as 0.5 mg/kg anti-VEGF yielded detectable levels in circulation up to 72 hours after injection (Fig. 7b). While treatment with only 0.5 mg/kg anti-VEGF or 5 mg/kg anti-JAG1 reduced CNV lesion size by ~53% and ~73%, respectively, combined treatment resulted in a ~86% decrease (Fig. 8b). This additive effect highlights Jagged1 as an attractive target for treatment of CNV.

### The effect of Jagged1 targeting is not dependent on antibody effector functions

Upon binding to target cells, monoclonal IgG antibodies can engage effector molecules, such as Fcγ receptors (FcγRs) and C1q. This may lead to activation of immune cells via effector functions that results in signaling events, phagocytosis or induction of cytotoxicity. Such effects may contribute to the therapeutic effect or be detrimental to ocular tissue in neovascular diseases, as has been observed in case of anti-VEGF therapeutics in AMD models and clinical settings^49–51^. In this study, we used mIgG2a antibodies, which are very potent inducers of Fc-mediated effector functions^52^. As such, we sought to determine whether the therapeutic effect was dependent upon effector functions.

To address this, we produced Fc-engineered variants of anti-VEGF and anti-JAG1 where three amino acid substitutions (P329G, L234A, and L235A (PGLALA)) were introduced in the CH2 domain of the Fc. These substitutions are expected to abolish binding to C1q and all FcγRs^53^. Following production in adherent human embryonal kidney (HEK)293E cells, size-exclusion chromatography (SEC) and SDS-PAGE analyses gave rise to pure monomeric fractions that bound recombinant mouse Jagged1 and VEGF in ELISA in a manner comparable to their WT counterparts (Supplementary Fig. 8) As expected for mIgG2a, the WT antibodies bound strongly to the four mouse FcγRs as well as the neonatal Fc receptor (FcRn), a regulator of IgG transport and plasma half-life^54,55^ (Supplementary Fig. 9). In contrast, the PGLALA-engineered antibodies did not bind the mouse FcγRs, but equally well to mouse FcRn (Supplementary Fig. 9).

Finally, we compared the PGLALA-engineered variants of anti-JAG1 and anti-VEGF in vivo with the WTs by IP injection (5 mg/kg) at D0 and D5 after laser treatment in the CNV mouse model (Fig. 8a). The PGLALA-engineered variants yielded a non-significant reduction of CNV size compared to WT anti-JAG1, in contrast to the ~threefold reduction for the anti-VEGF antibody (Fig. 8c). Thus, the therapeutic effect of Jagged1 blockade did not require immune effector functions.

## Discussion

It has been estimated that 5–15% of nAMD patients are non- or poor responders to current antibody-based anti-VEGF treatment and develop worsened visual acuity, or stationary or increased retinal edema despite monthly injections^56,57^. In addition, some patients develop treatment resistance over time^8^. While pathologically high levels of VEGF are a key factor, there are also other aspects of angiogenesis in CNV formation and exudation that drive the disease^58^. As such, other pro-angiogenic factors may be attractive targets in strategies to rescue loss of vision in patients that do not respond satisfactory to anti-VEGF. There are currently several strategies in the clinical pipeline aiming at providing new treatment options by combinatory targeting of angiogenic signaling^59,60^, and a bispecific IgG1 antibody harboring specificity for both VEGF and angiopoietin-2 (faricimab) was recently approved by the FDA to treat diabetic macular edema and AMD^15,61,62^. The rationale for targeting angiopoietin-2 is that its upregulation has been shown to destabilize endothelial cell layers, which leads to fluid leakage and makes the cells more responsive to VEGF^63^.

Here, we propose an alternative strategy to treat nAMD by specific blocking of the Notch ligand Jagged1 by a monoclonal antibody, either alone or in combination with anti-VEGF. We demonstrated the advantage of targeting Jagged1 by the use of an experimental laser-induced mouse CNV model, which upon anti-JAG1 treatment resulted in reduced inflammation, vascular growth, and CNV lesion size as well as immune cell recruitment and activation, which could relate to the activating role of Jagged1-Notch signaling on microglial phagocytosis^64^. Supported by in vitro cellular data, we also demonstrated that the anti-JAG1 effect was independent of VEGF. In addition, with no discernable effects in a non-angiogenic inflammatory model, and only modest reduction in cytokine and chemokine levels in the retina and the choroid-sclera, the underlying mechanisms of our findings are likely more related to inhibition of angiogenesis than inflammation We found that Jagged1 blockade did not alter Jagged1 expression, but led to upregulation of DLL4 in endothelial cells in CNV lesions. Because Jagged1 may act as a partial antagonist to DLL4^25^, Jagged1 blockade can augment DLL4-Notch1 signaling, which in turn increases DLL4 expression^65^. Hence, we postulate that the therapeutic effect of Jagged1 blockade could be at least in part explained by DLL4-mediated desensitization to pathological angiogenic signaling. Such a mechanism is in line with our cellular data, showing that stimulation of endothelial cells with DLL4 and VEGF results in reduced VEGFR2 expression, and with other demonstrations of DLL4 regulating angiogenic sprouting and inhibiting VEGF-induced endothelial cell responses^23,24^. While targeting of Jagged1 reduced CNV formation in vivo, the therapeutic effect was further enhanced by simultaneous administration of the anti-VEGF antibody. As such, anti-JAG1 may be given as monotherapy or as combinatory therapy together with anti-VEGF.

To our knowledge, this is the so far only report on Jagged1 targeting by a monoclonal antibody in the experimental CNV model. As expected, the effect was dependent on dosage of the anti-JAG1 and anti-VEGF antibodies. When the two antibodies were given at a high dose (5 mg/kg) alone, the effect of anti-VEGF treatment on CNV formation was more pronounced than when only anti-JAG1 was given. However, when we combined a 10-fold lower dose of anti-VEGF with a high dose of anti-JAG1, the treatment ultimately resulted in CNV size to near normal. The data suggest that Jagged1 antagonism may be a strategy to treat nAMD patients with treatment resistance to anti-VEGF monotherapy. Potentially, it may also be a relevant therapeutic target for other ocular diseases driven by neovascularization, but this remains to be investigated.

In contrast to our data, Ahmad et al. reported that the severity of laser-induced CNV in rats could be reduced by a small peptide corresponding to a region within the DSL domain of Jagged1^66^. This would indicate that Jagged1 signaling has the opposite effect than shown herein. However, since the effect of Jagged1 is to inhibit DLL4-Notch1 signaling through weakening the ability of DLL4 to activate Notch1^67^, this peptide may function as an activator of Notch rather than the ligand Jagged1 in situ. This view is supported by findings that a recombinant Notch1-trap to block Jagged1 reduces vascular permeability^47^, and has anti-angiogenic effects on both retinal and tumor angiogenesis^68^. Alternative strategies to target Jagged1-related effects in CNV could be either pan-Notch inhibition or by DLL4-mimicking peptides. However, pan-Notch inhibition by for example gamma secretase inhibitors may intensify the angiogenic response to laser injury^66,69^, and while DLL4-mediated Notch activation may inhibit CNV, myeloid Notch activation may actually drive CNV^70,71^. Thus, we assume Jagged1 to be a less ambiguous target than DLL4 and specific blocking through antibodies to be a suitable approach compared to pan-Notch strategies.

Reduced vascular leakage is a perhaps particularly important aspect of Jagged1 antagonism, as chronic edema causes permanent damage to retinal photoreceptors. Others have found Jagged1 blockade to increase extravasation in animals with dermal wounds^28^, and it may increase vascular permeability in other inflammatory conditions like contact dermatitis. Our findings that antibody blockade of Jagged1 reduced ear-swelling in the contact hypersensitivity model, as well as reduced vascular leakage in CNV as evaluated by FA, indicates an anti-leakage effect of Jagged1 blockade. Recent reports that Notch1-signaling prevents leakage supports this^72,73^. While we did not find Jagged1 blockade to reinstate the cellular junction proteins ZO-1 or VE-cadherin in CNV, the anti-leakage effect could be related to DLL4-mediated non-canonical Notch signaling or a decrease in VEGFR2 levels^72,73^. Others have found signaling through VEGFR2 to facilitate edema in models of diabetic retinopathy, which may relate to Jagged1-mediated signaling^47,74^. Notably, anti-JAG1 reduced retinal concentrations of the pro-inflammatory cytokine TNF-α after CNV induction, indicating an anti-inflammatory effect of Jagged1 blockade. TNF-α increases during CNV^75^, promotes angiogenesis and vascular permeability, and is known as a potent inducer of endothelial Jagged1 expression through NF-κB^25,76^. Thus, some of the ability of anti-JAG1 to attenuate CNV might be related to interruption of a feed-forward interaction between TNF-α and Jagged1.

The mouse anti-JAG1 mIgG2a antibody used (anti-JAG1.b70) binds strongly to the mouse ligand with a KD of 6 nM^35^. It fully discriminates between Jagged1 and Jagged2 and inhibits signaling through NOTCH1, −2 and −3. Importantly, the phage-display selected antibody also binds human Jagged1, and 7.5-fold stronger than the mouse counterpart (KD of 0.79 nM). Structurally, a solved co-crystal structure of the Fab fragment of specifically anti-JAG1.b70 in complex with a recombinant soluble form of human Jagged1 has revealed that it binds in an area located between the EGF-1 and Delta-Serrate-Lag DSL domains of Jagged1 (PDB structure 5BO1).

Observations that anti-VEGF antibody-based therapeutics, such as aflibercept and bevacizumab, in complex with VEGF may facilitate platelet aggregation and thrombocyte activation through FcγRIIa in both human and transgenic murine model systems, highlights the importance of considering Fc-mediated effector functions in an ocular setting^50,51^. As Jagged1 is membrane-bound, anti-JAG1 may cross-bind Jagged1 ligands and potentially initiate unwanted Fc-mediated side-effects by engaging of FcγR expressing immune cells. If the therapeutic outcome is solely dependent on blockade, and independent of effector functions, the constant Fc part can be engineered to be effector-negative. This will then reduce the risk of adverse events^15^. In our study, we addressed this by comparing the WT anti-JAG1 with an Fc-engineered version with three amino acid substitutions (PGLALA)^53^. Our results revealed that reduction of CNV size only requires blocking of Jagged1. As such, a humanized version could be made by combining anti-Jagged binding Fab with an effector function silenced Fc or an alternative scaffold. Notably, the PGLALA-substitutions do not affect the ability of the Fc to interact in a pH-dependent manner with FcRn, which regulates plasma half-life and transcellular transport^54,55,77^. Preventing the interaction with FcRn may however also be beneficial in a clinical setting to minimize systemic exposure following intravitreal injection^15,78^. Surprisingly, PGLALA anti-VEGF exhibited an increased therapeutic effect on lesion size compared to the WT antibody in our model. This suggests a possible effector-related impairment on anti-VEGF approaches, and is in line with PGLALA being present in the aforementioned faricimab.

Furthermore, our study centers on a mouse in vivo model where antibodies were administrated by IP injection. This is in contrast to a clinical setting where antibody-based therapeutics are given by intravitreal injections (IVTs), in which the aim is for the antibody design to retain high ocular concentrations while minimizing systemic exposure^78–80^. However, accurate interpretation and translation of pre-clinical studies must take into consideration how the therapeutics are administrated as well as cross-species differences that may affect the outcome^52,81–83^. One challenge is that IVTs in mice are notoriously difficult due to anatomical challenges. To translate our encouraging findings on Jagged1 targeting into clinical practice, the concept must be tested in established larger-animal models for laser-induced CNV and IVTs, preferably in non-human primates, which are extensively used for preclinical evaluation^15,84–87^.

## Methods

### Animals

Care, use, and treatment of experimental animals were in strict agreement with the ARVO Statement for the Use of Animals in Ophthalmic and Vision Research. Female C57BL/6 J mice (8–10 weeks old; Janvier Laboratory, Le Genest Saint Isle, France) were used in this study. Animal care and experiments were carried out in accordance with Norwegian and EU guidelines for care and use of laboratory animals, and were approved by the Norwegian Food Safety Authority (FOTS IDs 8620, 9129, 14191, and 28347). All animal studies were conducted at the Department of Comparative Medicine, Oslo University Hospital, Rikshospitalet. Female mice were used for all studies to avoid gender influence on outcome and variation of laser-induced CNV. Animal care and experiments were carried out in accordance with Norwegian and EU guidelines for care and use of laboratory animals, and were approved by the Norwegian Food Safety Authority (FOTS IDs 8620, 9129, 14191, and 28347). All animal studies were conducted at the Department of Comparative Medicine, Oslo University Hosptial, Rikshospitalet. Animals were kept in open cages (Eurostandard type III) that were changed every or every second week, depending of number of animals/cage, with a maximum of 8 adult animals per cage. All animals were provided with enrichment in the form of houses, tunnels, paper, and chewing sticks. The relative humidity in the housing environment was maintained at 55% ± 10%, with an air exchange rate in the housing rooms of 20/hour and a temperature of 21 ± 1 °C and a light cycle of 12 hours dark/12 hours light, with one hour gradually dusk and dawn from 7 am and 7 pm and complete darkness between 8 pm and 7 am. During the lit part of the light cycle, lighting strength was maintained at day standard (70–100 lux). Animals had free access to water (purified by reversed osmosis and ionic exchange) and food ad libitum. In all procedures, animals were anesthetized by IP injection of 100 mg/kg ketamine hydrochloride (Pfizer Animal Health, New York, NY) and 10 mg/kg xylazine (CP-Pharma, Burgdorf, Germany), and pupils were dilated with topical administration of tropicamide 5 mg/ml and phenylephrine hydrochloride 100 mg/ml (Bausch & Lomb UK Ltd., Kingston-upon-Thames, England).

### Antibody treatment

The anti-JAG1.b70^35^, anti-VEGF B20.4.1.1^88^, and the isotype control antibody (IgG, anti-Ragweed) were provided by Genentech. Anti-JAG1 (HMJ1-29, hamster IgG) mAb was provided by Professor Hideo Yagita, Juntendo University, Tokyo, Japan^37^. WT and PGLALA variants of anti-VEGF B20.4.1.1 and anti-JAG1.70 were produced in HEK293E cells as described below. All antibodies were diluted in sterile PBS and injected IP at a dose of 5 mg/kg body weight unless otherwise specified on the day of laser photocoagulation (day 0) and on day 5.

### Recombinant proteins and staining reagents

All commercially acquired proteins, their usage, concentrations, and suppliers used are listed in Supplementary Table 1.

### CNV Induction

Laser photocoagulation was performed using a 532-nm laser (Visulas 532 S; Carl Zeiss Meditec, Oberkochen, Germany). The pulses had a duration of 0,1 second, a power of 80 mW, and a spot size of 50 µm. Prior to laser pulse application, each eye was anesthetized with topical tetracain hydrochloride 10 mg/ml (Bausch & Lomb). Lubricating eye drops (Methocel 2%; Omnivision, Neuhausen, Switzerland) on a glass coverslip were applied to the cornea, and the retina was viewed through a slit lamp microscope. Each laser pulse (three or four per eye) was applied approximately two to three disc diameters from the optic disc. A vaporization bubble indicated disruption of Bruch’s membrane. After the application of laser burns, sterile eye drops (Viscotears; Laboratoires Thea, Clermont-Ferrand, France) was applied to both eyes. Mice were then placed on a pre-warmed plate at 35 °C until they awakened.

### Antigen binding and Fc receptor ELISA

96-well ELISA plates were coated with 500 ng/mL of either recombinant, soluble murine Jagged1 (or murine VEGF diluted in PBS and added at a volume of 100 µL per well. Following ON incubation at 4 °C, plates were blocked by adding 250 µL of PBS supplemented with 4% skimmed milk (M) and incubating at RT with shaking for 2 hours. All plates were washed four times with PBS supplemented with 0.05% Tween20 (T). Antibodies were diluted in PBSTM and added to the plates in a titration series from 2000–15.63 ng/mL in a final volume of 100 µL. After 1.5 hours of incubation on a shaker at RT, the plates were washed as previously described.

To verify antigen binding and protein functionality of the murine antibodies, the plates were washed as previously described and 100 µL of anti-murine Fc from goat conjugated to alkaline phosphatase (ALP) diluted in PSTM added to all wells. After final incubation (1 h, RT on a shaker) and washing, the ELISA was developed by adding 100 µL of 10 µg/mL *p*-nitrophenyl-phosphate substrate (S0942, Sigma Aldrich) dissolved in diethanolamine solution. Absorbance at 405 nm was measured on a Sunrise spectrophotometer (Tecan).

To evaluate Fcγ and neonatal Fc receptor (FcγR and FcRn) binding to the relevant antibodies, ELISA plates were coated with either 500 ng/mL murine VEGF or an equimolar amount of soluble murine Jagged1 (1923 ng/mL). Antibodies were added to blocked ELISA plates in a titration series from 2000 to 0.92 ng/mL. Next, biotinylated murine-soluble versions of FcγR1-4 (CD64, CD32, CD16, and CD16-2, Sino Biologics) and FcRn (Immunitrack; Supplementary Table 1) were incubated with ALP-conjugated streptavidin in 20 µL PBSTM (pH 7.4 and 5.5 for FcγRs and FcRn, respectively) at RT for 20 minutes and added to the ELISA at final concentrations of 250 ng/mL FcRs and 3.36 µg/mL streptavidin-ALP. After 1 h shaking incubation at RT, Fc-FcR binding was visualized by adding ALP-substrate.

### Half-life analysis

A titration series of anti-JAG1 and anti-VEGF (5.0, 0.5, and 0.05 mg/kg) was injected IP into 6 mice per dosage. Blood samples of 25 µL were drawn from the saphenous vein at 24, 48, 72, 120, 168, and 264 hours post injection through heparinized micro capillary pipettes. The samples were kept on ice and centrifuged at 17,000 × *g* at 4°C for 5 minutes to separate the serum from the plasma. The serum was harvested from the samples, diluted 1:10 in 50% glycerol/PBS and stored in 96-well plates at −20°C until analysis.

Analysis was carried out by antigen-binding ELISA. Serial dilutions of either anti-JAG1or anti-VEGF from 2000 to 0.98 ng/mL was added to each plate as a quantification standard. Serum samples were diluted 1:400–1:50 in 100 µL PBSTM and added to the blocked ELISA plates. Serum antibody levels were calculated by nonlinear regression and interpolating antibody levels against the standard in GraphPad prism (version 8.3.1, GraphPad Software LLC). Values were transformed to a logarithmic scale and calculated using a log(agonist) vs. response equation for variable slopes. Serum concentrations were calculated as a percentage relative to that found after the presumed end of the α-phase (= after 24 hours, set to 100%). β-phase half-life was calculated in Microsoft Excel version 16.16.19 (200210) using the equation *t*_1/2_ = log 0.5(log *A*_*x*_*/A*_*0*_)*t, where A_x_ is the amount calculated at the given time (*t*) and A_0_ is the amount calculated after 24 hours. Compound elimination was visualized by a nonlinear fit of the percentage values to a semilogarithmic model in GraphPad prism.

### Production of recombinant proteins

The cDNA sequences encoding the constant domains of WT mIgG2a isotypes and the variable heavy chains (HCs) of anti-JAG and anti-VEGF were synthesized and subcloned into pFuse vectors with zeocin resistance by GenScript Biotech Corporation. Matching murine kappa light chains (kLCs) were synthesized and subcloned into separate pFuse vectors. Effector-negative antibodies were generated by targeted mutagenesis of the constant domains to introduce PGLALA (P329G, L234A, and L235A)^53^.

Resulting constructs were used in transient co-transfection of HEK293E cells at a HC:LC ratio of 1:2 using Lipofectamine 2000 as per manufacturer’s instructions. Antibodies were collected as supernatant fractions every day for 10 days and further purified on an anti-murine kLC CaptureSelect affinity matrix (ThermoFisher, 09050907). Protein fractions were eluted by the use of 0.1 M glycine-HCl (pH 3.0) and neutralized by adding 1 M Tris-HCl (pH 8.0). Eluates were buffer exchanged into sterile-filtered PBS on 50 K spin columns (Merck Millipore, UFC905096) and monomeric fractions secured by size-exclusion chromatography (SEC) by the use of a Superdex200 10/300 column (GE Healthcare) coupled to an Äkta Avant 25 (GE Healthcare)*.* Resulting histograms and a non-reducing SDS-PAGE run on a 12% Bis-Tris polyacrylamide gel (ThermoFisher, NW00125BOX) were used to evaluate protein size and purity. Proteins were stored in LoBind Eppendorf tubes (Eppendorf, Z666505) at −20 °C until the day of experiments.

### Fundus fluorescein angiography (FFA)

FFA was performed with the retinal imaging microscope Micron IV (Phoenix Research Laboratories, Pleasanton, CA, USA). Laser treatment was performed as described above except the image-guided laser system (Meridian Merilas 532 green laser photocoagulator attached to the Micron IV) was used. Four laser burns were induced in each eye with a fixed spot size of 50 µm, duration of 70 ms, and power level of 270 mW. 10 days after laser photocoagulation, mice were anesthetized, pupils dilated, and IP injected with 10 mg/ml fluorescein sodium (Anatera; Alcon Laboratories, Fort Worth, TX, USA) at 100 µg/g body weight. The animals were positioned on the Micron IV stage and Hypromellose coupling fluid applied to the eye. The camera and eye position was adjusted ensuring correct alignment and focus on the optic nerve head plane using standard color fundus photography before adjusting to the appropriated filter set for fluorescein angiography. Images were captured using the Discover software (Phoenix Research Laboratories). Fluorescent fundus images were taken with the Micron IV light source at maximum intensity and without extra gain at 11, 13, and 15 minutes after fluorescein injection. The fluorescent area of CNV lesions was determined using open-source software, ImageJ (National Institutes of Health, Bethesda, MD, USA). Briefly, RGF Tiff files were imported into ImageJ and individual color channels were separated. Red and blue channels were removed and all area values were measured from the green channel. A green channel binary image was then generated using automated image thresholding (Otsu’s threshold clustering algorithm), CNVs were surrounded with the polygon selection tool and the fluorescent area for each CNV lesion was calculated.

### Murine contact hypersensitivity model

DNFB-induced contact hypersensitivity is a delayed-type hypersensitivity model for human allergic contact dermatitis. Mice were sensitized to DNFB by painting the shaved abdomen with 25 μl of a 0.5% solution in acetone/olive oil (4:1), in addition to 5 μl to each paw, on day 0 and 1. Treatment with Anti-JAG1.b70 was compared with isotype control—both at 5 mg/kg—by IP injections 3 hours before induction of the elicitation phase. The elicitation phase was induced on day 5 by painting the right ear with 20 μl of 0.5% DNFB solution. The thickness of the ears was measured using a micrometer. Swelling was calculated by subtracting the baseline thickness from the thickness measured at various time points. Anesthesia was induced by isoflurane inhalation. No animals were excluded from the analysis. Ears were harvested, fixated in formalin, and embedded in paraffin sectioned longitudinally, for later immunohistochemical analysis.

### Immunostainings and microscopy of retinal pigment epithelium (RPE)-choroid-sclera flat mounts

Mice were euthanatized 10 days after laser photocoagulation. Eyes were immediately enucleated and fixed with 4% paraformaldehyde (Sigma-Aldrich, St.Louis, MO, USA) in PBS for 1 hour at room temperature. After three washes with PBS, the anterior segment and retina were dissected out, four or five radial cuts in the remaining RPE-choroid-sclera were made, and incubated with a blocking buffer (PBS+ 0.5% Triton X-100 + 5% donkey serum) for 2 hours at 4 °C. After removal of the blocking buffer, the eye tissues were subsequently incubated with primary antibodies diluted in PBS with 1.25% BSA + 1% Triton X-100 overnight at 4 °C. After three washes with PBS, the tissues were incubated with fluorescently labeled secondary antibodies for 90 minutes at room temperature. Hoechst 33258 nuclear dye (0.5 µg/ml) was used as counterstain. The RPE-choroid-sclera complexes were then flat mounted with ProLong Diamond Antifade mountant (ThermoFisher) on a glass slide. Used antibodies are listed in Supplementary Table 1.

Fluorescence microscopy was performed with a Nikon Ellipse 80i (Nikon, Tokyo, Japan) with Nikon Plan Fluor oil objectives ×20/0.75 and ×40/1.30. Images were obtained using ZEN pro (Carl Zeiss Microscopy GmbH, Jena, Germany) image acquisition software. Single-channel images for each fluorochrome were captured at ×40 magnification. All digital images were taken under the same conditions. CNV areas (in µm^2^) were quantified by ICAM-2 immunostaining using ImageJ software. Briefly, CZI files were imported into ImageJ, and individual color channels separated. A binary image was then generated using automated image thresholding (Otsu’s threshold clustering algorithm), CNVs were surrounded with the polygon selection tool, and the ICAM-2-positive areas were calculated. To ensure reliable data analysis of the CNV lesions, exclusion criteria were applied as described previously^89^. Briefly, a lesion was excluded if (1) there was choroidal hemorrhage encroaching on the lesion; (2) the lesion was linear instead of circular; (3) the lesion had fused with another lesion; (4) the lesion had a size indicating it was an outlier lesion as defined below; or (5) the lesions was the only lesion in an eye. An outlier lesion was categorized as (1) “too big” (>10,000 µm^2^ in area and was more than five times larger than the next biggest lesion in the eye; (2) “too small” (<1/5 the area of the next smallest lesion in the eye; this criterion applied only if at least one lesion in the eye was more than 5000 µm^2^); or (3) “too different” (after all of the lesions in a specific treatment group were measured, the lesion’s area was fivefold greater than the mean for that group; this criterion applied only for lesions that were >5000 µm^2^.

Quantification of IBA-1-positive reactive mononuclear phagocytes in CNVs was performed as described previously^39^. Briefly, a 200 µm diameter circular selection around the laser spot was defined using ImageJ 1.53k and the number of round-shaped (ameboid) versus ramified mononuclear phagocytes counted. The built-in cell counter module and visual grid was applied in ImageJ to aid in counting, to mark and classify counted cells. Both the counting and morphology classifications were performed by two independent observers.

### Confocal microscopy image processing

All images were taken under the same conditions on a Zeiss LSM 880 confocal microscope, with tile scan and ×60/1.40-NA oil immersion objective. CZI files were imported into FIJI software and individual color channel separated. A binary image was then generated using automatic image thresholding (Otsu’s threshold clustering algorithm).

Pericyte coverage of retinal vessels was calculated from four images per retina. All digital images were taken under the same conditions. Pericyte coverage was calculated as the ratio of area covered by NG2 positive cells and normalized to the area covered by Isolectin B4 positive endothelial cells. CZI files were imported into ImageJ and individual color channels separated. A binary image was then generated using automated image thresholding (Otsu’s threshold clustering algorithm).

### Quantification of Fluorescence intensity

For fluorescence intensity quantification, the confocal images were opened using FIJI software. The images were stacked and sum slices were made in Fiji. After that, all channels were split, and threshold for each channel was calculated individually. The fluorescence intensity of target protein, such as DLL4 and Jagged1 were calculated in separate channels. Areas to calculate in each image were selected manually. The graphs show quantification of four replicates per group.

### Light-induced retinal degeneration

The model of fundus camera-delivered light-induced retinal degeneration was described recently in detail^41^. In brief, mice were dark-adapted overnight, anesthetized, and pupils dilated as described above. Fluorescein was administered as a single IP injection of 100 µl 20 mg/ml fluorescein sodium (total dose of 2 mg fluorescein/mouse). Mice were then placed on the animal stage; the optic disc was centered in the field of view and the camera focused on the retina. 3 minutes after the fluorescein injection, light was applied to the right eye at an intensity of 54,000 lux for 4 minutes. Light intensity from the Micron IV fundus camera was measured using a light meter (Cat # S90199, Thermo Fisher Scientific). After repositioning, the left eye was illuminated for 4 minutes starting 10 minutes after the fluorescein injection. Immediately after illumination of the 2nd eye, mice were injected IP with either anti-JAG1.b70 or isotype control antibody and placed on a pre-warmed plate at 35 °C until they awakened. 7 days after illumination, mice were euthanatized, eyes immediately enucleated, and fixed with 4% paraformaldehyde in PBS for 1 hour at RT. Right eyes were processed for retinal and RPE-choroid-sclera flat mounts, while left eyes were paraffin-embedded, sectioned, and stained with TUNEL assay.

### Terminal transferase dUTP nick end labeling (TUNEL)

Samples were fixed in 4% paraformaldehyde and embedded in paraffin, then sectioned accordingly. Rehydration and consequently permeabilization diluted Proteinase K 1/100 in dH2O was performed, followed by quenching or inactivation of endogenous peroxidases. After washing, the TUNEL assay was performed according to the manufacturer’s instructions (Abcam, USA; TUNEL Assay Kit-HRP-DAB (ab206386). The sections were then rinsed with PBS, counterstained with hematoxylin, and coverslipped before analysis by light microscopy. Stained slides were scanned by a Pannoramic MIDI II scanner (3DHISTECH, Hungary) at ×20 magnification and digitized with high resolution, at a pixel size of 0.243094 mm × 0.243094 mm.

### Quantification of cytokines and chemokines by Luminex technology

Total proteins from retinas (*n* = 6/group) or choroid-scleras (*n* = 12/group) were isolated using the cell lysis kit (171-304011, Bio-Rad Laboratories, Hercules, CA, USA) and lysis beads (Lysing Matrix D, 6913-100, MP Biomedicals) following the respective instructions. Protein extracts were quantified using the BCA method (Pierce BCA assay kit, 23225, Thermo-Scientific) and stored at −20 °C for maximum 1 week. All samples were normalized to the selected concentration of 1.3 mg/ml and assayed for cytokines. The cytokine/chemokine concentrations were determined using the Mouse Chemokine 31-plex Mouse (12009159, Bio-Rad Laboratories) according to the manufacturer’s instructions. Samples in duplicates were analyzed, using Bio-Plex MAGPIX Multiplex Reader and Bio-Plex Manager 6.1 software (Bio-Rad Laboratories).

### Cell culture

Umbilical cords were obtained from the Department of Gynecology and Obstetrics at the Oslo University Hospital, following the informed consent of donors, and according to a protocol approved by the Regional Committee for Research Ethics (S-05152). Human umbilical vein endothelial cells (HUVECs) were isolated as previously described^90^ and cultured in MCBD131 medium containing 7.5% FBS, 10 ng/mL EGF, 1 ng/mL bFGF, 1 µg/mL hydrocortisone, 50 µg/mL gentamicin, 250 ng/mL fungizone and 1% L-glutamine. Cells were maintained at 37 °C in 95% humidity/5% CO2 atmosphere, split 1:3, and used between passages 2 and 5. rhVEGF165 (Bio-Techne, Abingdon, UK) was added to confluent HUVEC cultures grown in 12-well plates at final concentrations of 0, 10, or 100 ng/ml, respectively, and cultured for another 24 hours. For serum starvation experiments, HUVECs were seeded in plates as previously specified, in serum-containing culturing medium. After 4 hours, cell attachment to the substrate was verified by visual inspection, before replacing the medium with serum-free medium (i.e., culturing medium with all supplements, except 7.5% FBS). Then, cells were incubated for 24 hours before starting experiments. Adherent HEK293E cells used for recombinant protein production were incubated at 37 °C and 5% CO_2_/95% air and cultured in GlutaMAX-containing RPMI medium (Life Technologies) supplemented with 10% fetal calf serum (FCS) and 25 U/mL penicillin (BioWhittaker). HEK293E cell cultures were split 1:5 between passages and grown to confluency prior to transfection.

### Notch stimulation with immobilized ligands

Plates were coated with recombinant human Jagged1 or DLL4 as previously described^91^. Briefly, for Jagged1, plates were coated with rabbit anti-His-tag in PBS for 1 hour at 37 °C, then blocked in complete medium for 1 hour at 37 °C before coating with recombinant human Jagged1 His-tag in PBS for 2 hours at 37 °C. For DLL4, plates were coated with anti-human IgG Fcγ in PBS for 1 hour at 37 °C, followed by blocking as previously described and coating with recombinant DLL4 Fc-tag for 2 hours. Cells were then seeded at a density of 2.5 × 10^4^/cm^2^ and incubated at 37^o^C overnight before stimulation with 10 ng/mL VEGF165 for 24 hours.

### Protein extraction and Western blot

Cells were washed with cold PBS and lysed in 10 mM Tris buffer (pH 6.8) containing 5 mM EDTA, 6 mM NaF, 5 mM tetrasodium pyrophosphate (Na_4_P_2_O_7_), 2% SDS, as well as inhibitors of proteases (Sigma P5726, 1:100) and phosphatases (Sigma P8340, 1:100). Sample buffer (72% glycerol, 28% β-mercaptoethanol, 0.33 mg/ml bromophenol blue) was added at a 1:7 ratio (v/v), and samples heated to 65 °C for 10 minutes. Protein concentrations were determined by the RC DC Protein Assay from Bio-Rad and 10 µg protein loaded in each well in a 15-well 10% or 4–20% polyacrylamide Bio-Rad mini-PROTEAN®TGX™ precast tris-glycine gel. 5 µl BLUltra Prestained Protein Ladder (6,5 to 270 kDa) (Bio-Helix) was used to estimate molecular weight of protein of interest. Separated proteins were transferred to a nitrocellulose membrane using the Trans-blot® Turbo™ transfer system using the program ‘mixed MW’. Following transfer, membranes were blocked with 5% no-fat milk (M) (Bio-Rad) or 5% BSA in TBST (Tris-buffered saline with 0.01% Tween 20, pH 7.4) for 30 minutes. Next, membranes were incubated with primary antibodies overnight (4 °C), followed by washing with TBST, and finally incubated with HRP-conjugated secondary antibodies for 2 hours. Substrate (SuperSignal™ West Dura Extended Duration Substrate, 32106 or 34076, Thermo Fisher Scientific) was added to the membrane, and product formation detected using ChemiDoc XRS+ system and Image Lab 4.1. When necessary, membranes were stripped using Restore PLUS western blot stripping buffer (Thermo Fisher Scientific) according to manufacturer’s instructions, then blocked again and stained with new antibodies. Anti-DLL4 was diluted in 5% BSA-TBST, anti-Jag1in 1% TBSTM, anti-β-tubulin in 1% TBSTM, and HRP-conjugated anti-rabbit IgG in 1% M as specified in Supplementary table 1. Bands were quantified using volume tools in Image Lab 4.1 and normalized to β-tubulin by using the following formula: (volume intensity protein of interest)/(volume intensity β-tubulin).

### Crystal violet proliferation assay

Relative cell density was determined as previously described by crystal violet staining^92^. Briefly, cells were seeded in 96-well plates at a density of 6 × 10^3^ cells per well in the presence of control IgG, bevacizumab, or anti-JAG1 antibodies at 25 µg/ml, and either with or without 10 ng/mL VEGF. Following cultivation for the number of days specified in results, cells were fixed using 4% paraformaldehyde for 10 minutes, then dried for 20 minutes then stored at 4 °C until the end of the experiment. Fixed cells were stained with 70 µl 0.1% crystal violet (Apotekforeningen, Oslo) in PBS for 4 minutes then thoroughly washed in cold tap water. Nuclear dye was then resuspended in 33% acetic acid (Sigma-Aldrich) on a shaking incubator at RT for 5 minutes before absorbance was measured at 550 nm in an Epoch (BioTek) plate reader with Gen5 software (BioTek).

### LDH assay

Cytotoxicity was measured by leakage of LDH using the Cytotoxicity Detection Kit^PLUS^ (LDH) (Sigma 4744926001) according to the manufacturer’s instructions. Briefly, cells were cultured for 3 days in medium supplemented with 10 ng/ml VEGF in the presence of 25 µg/ml control IgG, anti-JAG1, or bevacizumab. 100 µl of the detection reagent was then added to each well and incubated in the dark for 25 minutes before the stop reagent was added. OD was then measured at 492 nm with an Epoch (BioTek) plate reader with Gen5 software (BioTek).

### Immunohistochemistry of normal human eyes

Formalin-fixed, paraffin-embedded human eyes that either showed signs of dry AMD such as drusen formation and RPE alterations, or with no pathological traits, were obtained from the diagnostic biobank at the Division of Pathology, Oslo University Hospital, and used in accordance with a protocol approved by the Regional Committee for Research Ethics, Health Region South, Norway (approval 2013/803) following informed consent of all donors. JAG1 was detected by manual staining: tissue sections (4 µm thick, formalin-fixed, paraffin-embedded) were deparaffinized, boiled for 20 minutes in Dako Target Retrieval Solution pH 6, incubated with Dako Peroxidase Blocking Reagent for 5 minutes followed by incubation with rabbit monoclonal anti-JAG1 diluted in PBS with 1.25% BSA overnight at 4 °C, then incubated with Rabbit-on-Rodent HRP-Polymer (Biocare Medical, Pacheco, CA, USA) for 30 minutes followed by incubation with Discovery Purple chromogen for 30 minutes (Roche Tissue Diagnostics—Ventana Medical Systems, Tucson, AZ, USA). Hematoxylin was used as counterstain. Unless otherwise indicated, all incubations were performed at room temperature.

### Statistics

Data are presented as mean ± SEM unless otherwise specified. Unpaired students’ *t* test was used to compare experimental parameters to the relevant controls. *T* tests were performed using Prism 8 (GraphPad, San Diego, CA). Two-tailed *p* values ≤ 0.05 were considered statistically significant. For comparing multiple variables, one-way ANOVA with subsequent Bonferroni’s multiple comparison test was used.

### Reporting summary

Further information on research design is available in the Nature Portfolio Reporting Summary linked to this article.

## Supplementary information

Supplementary Information File

Reporting Summary

## Data Availability

All data supporting the findings described are available in this paper and in the Supplementary Information. The Source Data and Supplementary Information are provided in this article. All performed statistical analyses are described in the Source Data. In certain cases, representative microscopy images are shown for simplicity. Where specified, these and related images have been used for quantification purposes and statistical analysis. In these cases, related numerical data from both representative images and all related, not shown images are included in the Source Data provided with this article. Related, underlying raw image files are stored locally at the research institutions and may be obtained from the corresponding authors J.T.A. and E.S. following requests sent to email addresses provided with this paper (j.t.andersen@medisin.uio.no and eiriksundlisater@gmail.com) who will strive to handle requests within 60 working days. Source data are provided with this paper.

## Source data

Source Data
