# Supplementary material for: Antibody blockade of Jagged1 attenuates choroidal neovascularization
Source: Nat Commun. 2023 May 30;14:3109. doi: 10.1038/s41467-023-38563-w (PMC10229650; doi:10.1038/s41467-023-38563-w)
Supplement: Supplementary file 1 — Supplementary Information File [file 41467_2023_38563_MOESM1_ESM.docx]

**Antibody blockade of Jagged1 attenuates choroidal neovascularization**

*Supplementary Information*

**Supplementary figure 1: VEGF does not affect Jagged1 expression in HUVECs under normal growth conditions. (a-d**) HUVECs cultured for 48 hours before treatment with increasing concentrations of human VEGF and/or bevacizumab or an isotype control antibody for 24 hours. (**a** **and c**) Representative Western blots showing expression of Jagged1, DLL4 and β-tubulin under the specified conditions. (**b and d**) Quantification in ImageLab 4.1 of bands exemplified in (a) and (c). Values were normalized against β-tubulin. Data are means ±SEM representing measurements from 3 individual experiments in (b) and 2 individual experiments in (d). Source data are provided as a Source Data file.


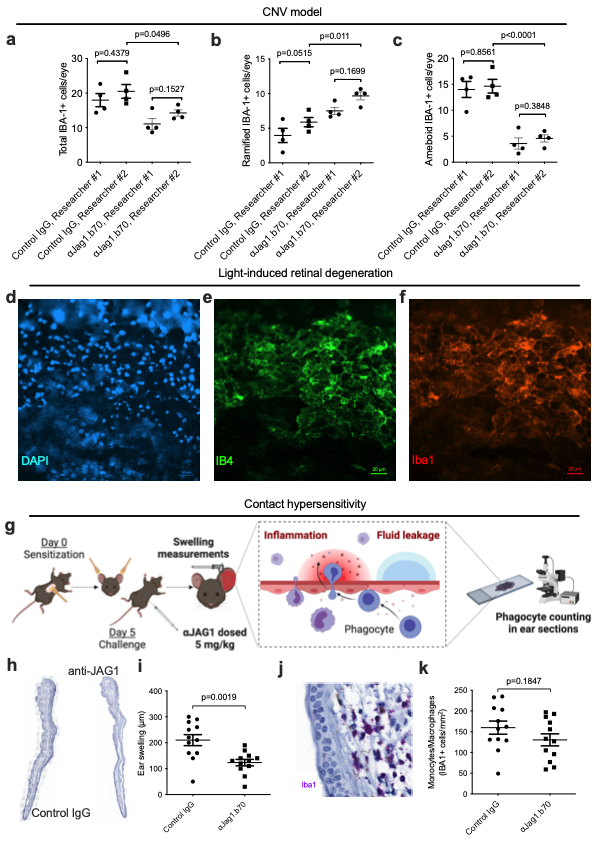


**Supplementary Figure 2.** **Additional data from inflammatory models. (a-c)** Comparison of monocyte analysis performed by two independent researchers on images obtained from RPE/choroidal sclera flat-mounts following CNV induction. Researcher #1 corresponds to data shown in figure 3d. Values show mean ± SEM (n=4 eyes from 4 individual mice per column; calculated from a total of 12 and 13 CNV lesions for control IgG and anti-JAG1, respectively). Indicated p-values results from two-tailed, unpaired Student’s t-test. **(d-f)** Images showing visualization of cells in eye sections following LIRD, showing (d) nuclei in blue, and exemplifying that IB4 (e) and Iba1 (f) staining yielded near identical cell identification. **(g)** Illustration of methodology of DNFB-induced contact hypersensitivity resulting in ear inflammation and increased vascular permeability. Following sensitization to DNFB at day 0, antibody treatment was administered at the same day as DNFB challenge (day 5). Resulting inflammation from DNFB challenge was measured as ear swelling and phagocyte counting in ear sections. The illustration was created with Biorender.com. (**h**) Ear sections from mice at end of experiment illustrated in (g) after systemic treatment with either anti-JAG1.b70 or control IgG. **(i)** Ear thickness at end of experiment, from ears exemplified with sections in (h). **(j)** Immunohistochemical staining with anti-IBA-1 antibody in an inflamed mouse ear. Values in (a-c), (i) and (k) show mean ± SEM and results from 6 individual mice per treatment group. Data were compared by two-tailed, unpaired Student’s t-test. Source data are provided as a Source Data file.

**Supplementary Figure 3.** **Concentrations of proinflammatory cytokines and chemokines in the retina or in the choroid-sclera.** The concentrations of 31 cytokines and chemokines were determined in the **(a-b)** retina or **(c-d)** in the choroid-sclera with a multiplex assay 4 days after CNV induction. Data are divided based on detected amounts (higher levels in a and c, lower in b and d) to clearly visualize all detected cytokines. Treatment with control-IgG or anti-JAG1 was administered by IP injection immediately after laser injury. Data are means ±SEM of duplicate determinations for two pooled samples (three retinas or six choroid-scleras were pooled as one sample). (a and b) CXCL13, CXCL5, CCL11, CCL24, GM-CSF, IFNγ, IL-4, IL-10, CXCL11, CCL2, CCL20, CCL5 and CCL17 were below detection limits. (b) CCL17 were below detection limits. Source data are provided as a Source Data file.

**Supplementary Figure 4.** **Retinal vascular morphology and endothelial cell proliferation is not affected by** **antibody targeting of Jagged1. (a-c)** Representative images of retinal flat-mounts stained with isolectin B4 at day 7 for each group. **(d-f)** Resulting images after analysis with AngioTool software. **(g-l)** Graphical representation of the AngioTool analysis performed on retinal flat-mount images from 4 control IgG-, 4 anti-Jag1.b70- and 4 anti-VEGF-treated mice. 3 images at 20x magnification from each retina were analyzed. Values show mean ± SEM. **(m-n)** Analysis of HUVEC proliferation by crystal violet staining in cultures with either control IgG, anti-JAG1.b70 or anti-VEGF (bevacizumab) following cultivation in either the presence (m) or absence (n) of VEGF. (**o**) Analysis of cell death in HUVEC cultures containing either control IgG, anti-JAG1.b70 or anti-VEGF (bevacizumab). Data are means ±SEM representing measurements from 3 individual experiments. Data were compared by 1 way ANOVA with Šídák's multiple comparisons test). Source data are provided as a Source Data file.


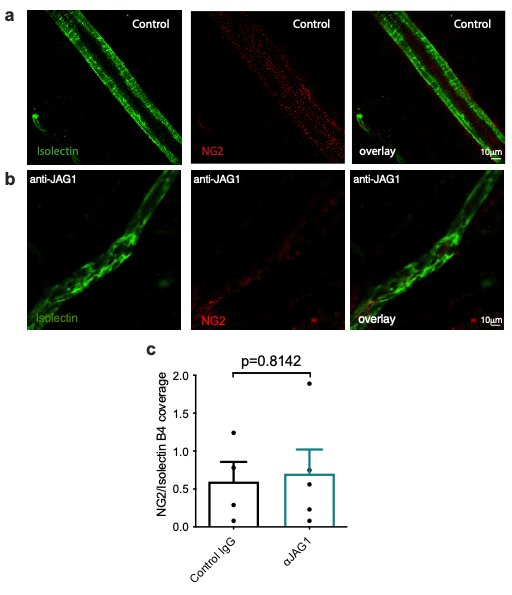


**Supplementary Figure 5.** **Pericyte coverage in retinal vessels is not affected by antibody targeting of Jagged1. (a-b)** Representative images of retinal flat-mounts stained with isolectin B4 (green) and NG2 (red) at P7 after treatment with (a) control IgG or (b) anti-Jag1.b70. (**c)** Quantification of pericyte coverage (ratio of total area of NG2 positive pericytes to isolectin-B4-positive capillaries) in fluorescent images of retinal flat-mounts treated with either control IgG (4 eyes from 4 individual mice) or anti-Jag1.b70 (5 eyes from 5 individual mice). Values show mean ± SEM (two-tailed, unpaired Student’s t-test). Source data are provided as a Source Data file.

**Supplementary Figure 6.** **Adherens junctions in CNV lesions at D10. (a-c)** Confocal images of flat-mounted RPE-choroid-sclera complexes from mice treated with either (a) control IgG, (b) anti-Jag1.b70 or (c) anti-VEGF, and labeled with antibody specific for ICAM-2 (green; marking endothelial cells) and VE-cadherin (red; marking adherens junctions). Immunostaining procedures yielding representative images shown in (a-c) were performed twice in individual experiments.

**Supplementary Figure 7.** **Tight junctions in CNV lesions at D10. (a-c)** Confocal images of flat-mounted RPE-choroid-sclera complexes from mice treated with either (a) control IgG, (b) anti-Jag1.b70 or (c) anti-VEGF, and labeled with antibody specific for ICAM-2 (green; marking endothelial cells) and ZO-1 (red; marking adherens junctions). Immunostaining procedures yielding representative images shown in (a-c) were performed twice in individual experiments.


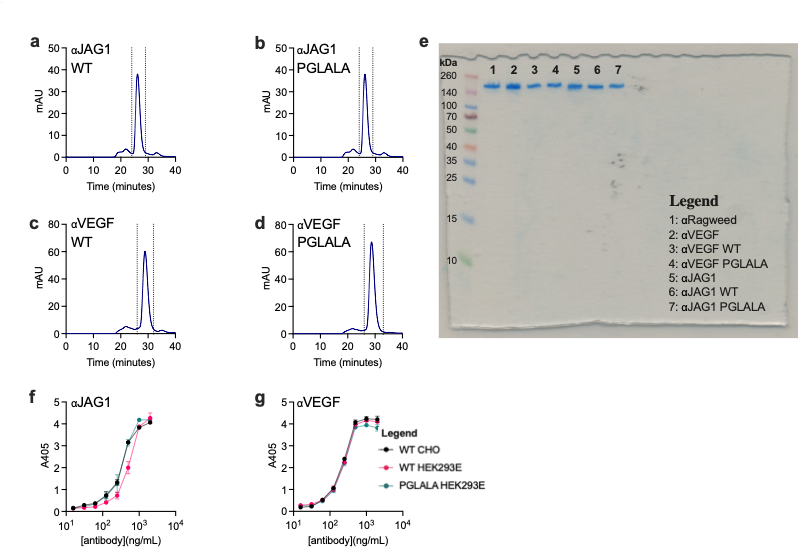


**Supplementary Figure 8. Purity and functionality of mouse anti-JAG1 and anti-VEGF mIgG2 variants.** (**a-d**) Monomeric fractions of recombinantly produced mIgG2a antibodies were isolated from peaks marked with stapled lines in obtained from size-exclusion chromatography using a Superdex200 10/300 column on an Äkta Avant25. (**e**) Subsequent SDS-PAGE gel showing the purified antibodies and the related isotype control (anti-Ragweed). Anti-VEGF/JAG1 without suffix denotes proteins obtained from Genentech. Wells were loaded with equal amounts of protein (~2 µg). The image has not been cropped. SDS-PAGE analysis was performed once to confirm purity observed in size-exclusion chromatography. (**f-g**) Binding of the WT and Fc-engineered antibodies to (f) mouse truncated Jagged1and (g) mouse VEGF in ELISA. The shown data represents mean values ± SD resulting from duplicate titration curves. Source data are provided as a Source Data file.


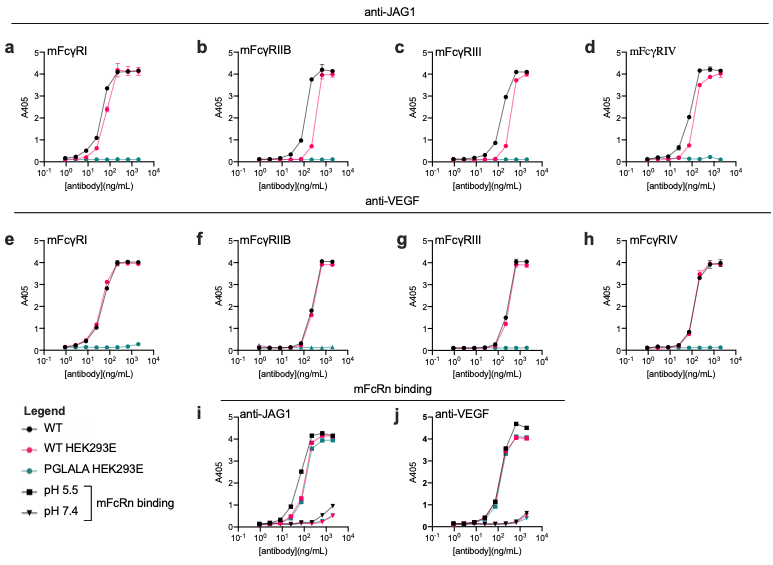


**Supplementary Figure 9. Binding of WT and Fc-engineered versions of anti-JAG1 and anti-VEGF to the mouse** Fcγ **receptors.** Binding of all used mIgG2a anti-JAG1 (**a-d**) and anti-VEGF (**e-h**) antibodies to recombinant forms of the four mouse FcγRs (shown data represents mean values ± SD resulting from duplicate titration curves), and mouse FcRn (**i** and **j**) upon capturing on their cognate antigens in ELISA. 2000-0.92 ng/mL antibodies were captured on equimolar amounts of the antigens. Binding to mouse FcRn was performed with buffers at both pH 5.5 and 7.4 using singular titration curves. Source data are provided as a Source Data file.

**Supplementary table 1: Overview of commercially acquired proteins and their usage**

| **Protein** | **Supplier** | **Cat.nr** | **Usage** | **Dilution/concentration** | **Validation in the relevant species / Reference(s)** |
| --- | --- | --- | --- | --- | --- |
| Anti-Jagged1 HMJ1-29 mIgG2a | Professor Hideo Yagita | - | *In vivo* studies | 1 mg/mL | PMID: 18381350 |
| Anti-Jag1.b70 mIgG2a | Genentech |  | *In vivo* studies | 1 mg/mL | PMID: 26580007 |
| Anti-mouse VEGF B20.4.1.1 mIgG2a | Genentech |  | *In vivo* studies | 1 mg/mL | PMID: 17360669 |
| Anti-Ragweed mIgG2a | Genentech |  | *In vivo* studies | 1 mg/mL | PMID: 26580007 |
| Murine CD64 | Sino Biologics | 50086-M27H-B | ELISA | 0.25 µg/mL | https://www.sinobiological.com/recombinant-proteins/mouse-cd64-50086-m27h-b |
| Murine CD32 | Sino Biologics | 50030-M27H-B | ELISA | 0.25 µg/mL | https://www.sinobiological.com/recombinant-proteins/mouse-cd32b-fcgr2b-50030-m27h-b |
| Murine CD16 | Sino Biologics | 50326-M27H-B | ELISA | 0.25 µg/mL | https://www.sinobiological.com/recombinant-proteins/mouse-cd16-fcgr3-50326-m27h-b |
| Murine CD16-2 | Sino Biologics | 50036-M27H-B | ELISA | 0.25 µg/mL | https://www.sinobiological.com/recombinant-proteins/mouse-fcgr4-50036-m27h-b |
| Murine FcRn | Immunitrack | ITF07 | ELISA | 0.25 µg/mL | https://www.immunitrack.com/eur/webshop/fcrn/murine-fcrn-murine-b2m.html |
| Murine Jagged1 | Abcam | AB109346 | ELISA | 0.50 µg/mL  and 1.90 µg/mL | https://www.abcam.com/products/proteins-peptides/recombinant-mouse-jagged1-protein-fc-chimera-active-ab109346.html |
| Murine VEGF-164 | Sigma-Aldrich | V4512 | ELISA | 500 ng/mL | https://www.sigmaaldrich.com/NO/en/product/sigma/v4512 |
| Goat anti-murine Fc-ALP | Sigma-Aldrich | A2429 | ELISA | 0.50 µg/mL | https://www.sigmaaldrich.com/NO/en/product/sigma/a2429 |
| Anti-DLL4 | Cell Signaling | 2589 | Western Blot | 1:1000 | <https://www.cellsignal.com/products/primary-antibodies/dll4-antibody/2589>  PMID: 35737588 |
| Anti-Jagged1 | Cell Signaling | 2155 | Western Blot | 1:1000 | [https://www.cellsignal.com/products/primary-antibodies/jagged1-1c4-rabbit-mab/2155](https://www.cellsignal.com/products/primary-antibodies/jagged1-1c4-rabbit-mab/2155%20/)  PMID: 34059639 |
| Anti-β-tubulin | Abcam | AB6046 | Western Blot | 1:20000 | https://www.abcam.com/products/primary-antibodies/beta-tubulin-antibody-loading-control-ab6046.html  PMID: 24018888 |
| Rabbit IgG-HRP | Jackson ImmunoResearch Laboratories | 711-035-153 | Western Blot | 1:20000 | https://www.jacksonimmuno.com/catalog/products/712-035-150  PMID: 33979025 |
| Anti-VEGFR2 | Cell Signaling | D5B1 | Western Blot | 1:1000 | <https://www.cellsignal.com/products/primary-antibodies/vegf-receptor-2-d5b1-rabbit-mab/9698>  PMID: 30949450 |
| Rabbit anti-His tag | GenScript | A00174-40 | Coating wells in cell stimuli experiments | 6.50 µg/mL | <https://www.genscript.com/antibody/A00174-His_tag_Antibody_pAb_Rabbit.html>  PMID: 22809957 |
| Goat anti-human IgG Fcγ | Sigma Aldrich | I2136 | Coating wells in cell stimuli experiments | 6.50 µg/mL | https://www.sigmaaldrich.com/NO/en/product/sigma/i2136 |
| Recombinant human Jagged1 (His-tag) | R&D Systems | 1277-JG | Coating wells in cell stimuli experiments | 2.50 µg/mL | https://www.rndsystems.com/products/recombinant-human-jagged-1-fc-chimera-protein-cf_1277-jg |
| Recombinant human DLL4 (Fc-tag) | R&D Systems | 1506-D4 | Coating wells in cell stimuli experiments | 1.70 µg/mL | https://www.rndsystems.com/products/recombinant-human-dll4-his-tag-protein_1506-d4 |
| Rat anti-mouse ICAM-2 | BD Biosciences | 553326 | Fluorescence microscopy | 1.30 µg/mL | <https://www.bdbiosciences.com/en-no/products/reagents/flow-cytometry-reagents/research-reagents/single-color-antibodies-ruo/purified-rat-anti-mouse-cd102.553326>  PMID: 18326747 |
| Rabbit anti-Jagged1 | Abcam | EPR4290 | Fluorescence microscopy, immunohistochemistry | 7.50 µg/mL | <https://www.abcam.com/products/primary-antibodies/jagged1-antibody-epr4290-ab109536.html>  PMID: 29378174 |
| Cy3-conjugated donkey anti-rabbit IgG | Jackson ImmunoResearch Laboratories | AB_2307443 | Fluorescence microscopy | 1.30 µg/mL | <https://www.jacksonimmuno.com/catalog/products/711-165-152> |
| Alexa Fluor 488-conjugated donkey anti-rat IgG | Life Technologies | A48269 | Fluorescence microscopy | 5.00 µg/mL | https://www.thermofisher.com/antibody/product/Donkey-anti-Rat-IgG-H-L-Highly-Cross-Adsorbed-Secondary-Antibody-Polyclonal/A48269 |
| Goat anti-Jagged1 | Santa Cruz Biotechnology | sc-6011 | Fluorescence microscopy | 3.00 µg/mL | <https://www.scbt.com/p/jagged1-antibody-c-20>  PMID: 22809957, 25100656 |
| Goat anti-DLL4 | R&D Systems | AF1389 | Fluorescence microscopy | 4.0 µg/mL | <https://www.rndsystems.com/products/mouse-dll4-antibody_af1389?gclid=CjwKCAiAu5agBhBzEiwAdiR5tDO3U3HNcT6MsJr-VlB5QtXxGPWuEkWoPnzBYZ1KC4r62Id8Cm9sfBoC6IYQAvD_BwE&gclsrc=aw.ds>  PMID: 28695891, 34910918 |
| Rabbit anti-NG2 | Sigma-Aldrich | ab5320 | Fluorescence microscopy | 5.00 µg/mL | <https://www.sigmaaldrich.com/NO/en/product/mm/ab5320>  PMID: 26501235 |
| Rabbit anti-IBA-1 | Abcam | Ab178847 | Fluorescence microscopy | 0.60 µg/mL | <https://www.abcam.com/products/primary-antibodies/iba1-antibody-epr16589-ab178847.html>  PMID: 33732230 |
| IB4-Alexa488 | ThermoFisher Scientific | i21411 | Fluorescence microscopy | 10.00 µg/mL | <https://www.thermofisher.com/order/catalog/product/I21411>  PMID: 17036005 |
| Goat anti-VE-cadherin | R&D Systems | AF1002 | Fluorescence microscopy | 3.00 µg/mL | <https://www.rndsystems.com/products/mouse-ve-cadherin-antibody_af1002?gclid=CjwKCAiAu5agBhBzEiwAdiR5tM9sxyIRdiO95ohTCGrCDnMITLd52rNEHBl3RmkhYwAOK6mUXusKKRoCJKQQAvD_BwE&gclsrc=aw.ds>  PMID: 29263363, 35864120 |
| Rabbit anti-ZO-1 | Abcam | ab221547 | Fluorescence microscopy | 3.00 µg/mL | <https://www.abcam.com/products/primary-antibodies/zo1-tight-junction-protein-antibody-epr19945-296-ab221547.html>  PMID: 32518142 |
| Alexa Fluor 546-conjugated donkey anti-goat IgG | Invitrogen | A11056 | Fluorescence microscopy | 5.00 µg/mL | https://www.thermofisher.com/antibody/product/Donkey-anti-Goat-IgG-H-L-Cross-Adsorbed-Secondary-Antibody-Polyclonal/A-11056 |
| Alexa Fluor 488-conjugated donkey anti-rabbit IgG | Invitrogen | A21206 | Fluorescence microscopy | 2.50 µg/mL | https://www.thermofisher.com/antibody/product/Donkey-anti-Rabbit-IgG-H-L-Highly-Cross-Adsorbed-Secondary-Antibody-Polyclonal/A-21206 |
